# Data science and automation in the process of theorizing: Machine learning’s power of induction in the co-duction cycle

**DOI:** 10.1371/journal.pone.0309318

**Published:** 2024-11-04

**Authors:** Daan Kolkman, Gwendolyn K. Lee, Arjen van Witteloostuijn

**Affiliations:** 1 Jheronimus Academy of Data Science, ’s-Hertogenbosch, the Netherlands; 2 Utrecht University, Utrecht, the Netherlands; 3 University of Florida, Gainesville, Florida, United States of America; 4 Vrije Universiteit Amsterdam, Amsterdam, the Netherlands; 5 Antwerp Management School, Antwerpen, Belgium; East China Normal University, CHINA

## Abstract

Recent calls to take up data science either revolve around the superior predictive performance associated with machine learning or the potential of data science techniques for exploratory data analysis. Many believe that these strengths come at the cost of explanatory insights, which form the basis for theorization. In this paper, we show that this trade-off is false. When used as a part of a full research process, including inductive, deductive and abductive steps, machine learning can offer explanatory insights and provide a solid basis for theorization. We present a systematic five-step theory-building and theory-testing cycle that consists of: 1. Element identification (reduction); 2. Exploratory analysis (induction); 3. Hypothesis development (retroduction); 4. Hypothesis testing (deduction); and 5. Theorization (abduction). We demonstrate the usefulness of this approach, which we refer to as co-duction, in a vignette where we study firm growth with real-world observational data.

## Introduction

In the social sciences, theory is oftentimes said to explain (or predict) an outcome by specifying the underlying causal mechanisms through which *A* causes *B*, perhaps mediated or moderated by *C* (e.g., [1, 2]). Developing explanations (or predictions) of an outcome, however, is particularly challenging for complex phenomena where many theories offer a large variety of explanations, but where the outcome still has a lot of its variance unexplained. A large variance unexplained may point to the dominance of ‘true’ randomness, or to current theoretical approaches falling short of explaining the outcome well, as well as current statistical models of the outcome failing to achieve convincing predictive power. The central aim of the current paper is to offer a methodology that combines different reasoning approaches and empirical techniques to facilitate the search for better theories. Here, theories are better if they (a) reduce unexplained variance (of phenomena not associated with dominant randomness) whilst (b) identifying plausible underlying causal mechanisms. This is easier said than done, as both objectives can be at odds with one another. If so, the (b) criterion should receive more weight *vis-à-vis* (a).

Specifically, we integrate modern machine learning (ML) and classic regression techniques in a methodological cycle that combines induction and deduction with abduction in the context of a systematic research process pipeline. We are not the first to promote the use of ML in the social sciences, but we move beyond what has been contributed so far. Recently, for example, algorithm-assisted theorizing has been advocated as a new approach for theory building in management research and organization science. For instance, pattern detection supported by ML algorithms has been proposed to facilitate inductive theorizing [3, 4], exploratory pattern discovery has been suggested to assist data-driven inductive theory building and to detect patterns that may have gone unnoticed in post-hoc analysis of regression results [5], and ML has been applied to provide better predictions in the context of policy [6]. However, such pleas for algorithm-assisted research emphasize the usefulness of pattern exploration, but do not detail how ML can contribute to theory development in the context of a systematic research process pipeline that integrates the use of ML with that of classical methods. Precisely this is what we seek to do in the current paper.

In so doing, our contribution is in the spirit of the argument that Van Lissa [7] recently made in the context of developmental psychology. As we do, he argues that “theory formation requires inductive (exploratory) research. This paper argues that machine learning can help advance theory formation … because it enables rigorous exploration of patterns in data” (p. 1). Our key contribution is that we further develop this argument by suggesting a concrete and systematic research process pipeline that combines ML with classical methods, and that we illustrate the working of this pipeline with observational data regarding firm growth. The research process pipeline, or ‘co-duction cycle’, that we propose here provides a stepwise approach aimed at theory formation that cumulatively increases our understanding of complex phenomena. The critical role of ML is to identify **unknown** complexities in data through automated pattern detection that subsequently function as the building blocks of new theory, which can then be tested by applying classical methods.

The emphasized word ‘unknown’ here is critical. The classical methods in the tradition of probabilistic statistics we are all so familiar with require that the researcher specifies a data generation process in advance; ML does not. Basically, ML techniques are automated algorithms that combine many—oftentimes weak—determinants to learn the data generation process that generates maximal—or rather, optimal (i.e., penalizing for overfitting)—model fit in the form of predictive accuracy [8]. Importantly, this is done without *a priori* specifying a model associated with specific relationships (main, mediation or moderation, of whatever order) or functional forms (linear of non-linear). In the social sciences, we are rarely aware up-front of the many subtle complexities involved in the data generation processes we study. Hence, we tend to have a very hard time specifying all these complexities in advance, as is required when we apply our classical methods—mostly variants of the linear probabilistic regression model. This is where ML comes in. By applying ML first, we can identify the key complexities that characterize the data generation process under study that we can then use as input for theory formation embracing these identified complexities, which can subsequently be tested with classical methods (see [9]).

The key yardstick in ML is predictive accuracy (after balancing over and underfitting). Predictive power is a key criterion for evaluating an inference to a plausible explanation, as posited in the philosophy of statistics [10, 11]. When the current theories fail to explain much of the variance in the outcome that these current theories seek to explain, this not being due to ‘true’ randomness, we need a methodology for theory building that aims to increase explanatory and predictive power. We suggest a methodology that combines the strengths of ML with those of classic regression, compensating for each other’s weaknesses. The methodology we propose synergistically (and sequentially) integrates modern ML techniques and classic probabilistic regression with the aim to facilitate theory building. Along the way, as a by-product, we expand the metrics and visualizations associated with the ML technique known as Random Forest Analysis in order to further open the black box of this much-used ML algorithm. In so doing, Random Forest Analysis’s output can be meaningfully interpreted in order to inform the next regression stage in the theory-building cycle. We note that the ‘co-duction cycle’ might be relevant for any discipline studying complex phenomena by analyzing observational data.

We illustrate our new theory-building methodology with real-world observational data of high dimensionality (i.e., including many variables) that are much more complex than the artificial data that are created with a pre-set data generating process (cf. [4, 5], but see [9] Choudhury et al., 2021; Shrestha et al., 2021; but see Bosma & van Witteloostuijn, 2024). Indeed, high dimensionality is common in empirical research in the social sciences. This is why we decided to face the messiness of observational data head-on. We demonstrate the use of our proposed methodology with observational data about a classic theoretical and empirical puzzle: firm growth. After close to a century of research since Gibrat [12] started the inquiry, by and large, the theory of firm growth is still far from complete [13]. The literature has accumulated a large number of theories that seek to explain why some firms grow at a higher rate than others, and some do not do so at all (or fail altogether), with a wide variety of research methods having been employed to explain and predict the rate of firm growth. Yet, empirical findings have reported persistently a high degree of unexplained variance.

A literature review by Coad [14] (p. 12) concludes that empirical work seeking the determinants of firm growth has made limited progress; “the combined explanatory power of the explanatory variables (summarized by the R^2^ statistic) is typically low, usually below 10 per cent.” Statistical models with high model fit for the rate of firm growth have been elusive [15]. More recent research points out that, at least in part, firm growth may be approximated by a random walk [16], can be seen as the result of mere luck [17] or emerges as a chaotic potpourri of different paths [18]. This current state of affairs may simply be the end point, as the ‘true’ data generation process underlying firm growth might well have a very dominant random component. However, alternatively, it may be that extant research has not yet been able to identify essential complexities. Clearly, the firm growth literature reflects a typical example of a research puzzle where all of the (combinations of) many theoretical perspectives are associated with much unexplained variance, making this case ideal for illustrating the value added of the new co-duction methodology that we do propose in the current paper.

The rest of the paper is organized as follows. After setting the scene by summarizing the background of scientific methods for building theory with observational data, we present a cycle of what we refer to as one involving ‘*co-duction*’ methods—*reduction*, *induction*, *retroduction*, *deduction*, and *abduction*. The cycle builds on the methodologies that are advocated by Judea Pearl [19] and Herbert Simon [20], both being pioneers of artificial intelligence. We note that Judea Pearl received the A. M. Turing Award in Computer Science in 2011 “[f]or fundamental contributions to artificial intelligence through the development of a calculus for probabilistic and causal reasoning” and Herbert Simon received the Sveriges Riksbank Prize in Economic Sciences in Memory of Alfred Nobel in 1978 “for his pioneering research into the decision-making process within economic organizations.” Simon and co-recipient Allen Newell of the 1975 A. M. Turing Award made fundamental contributions to artificial intelligence.

After presenting the co-duction cycle, we then demonstrate the use of the methods with a vignette in which we extend earlier empirical work [21] that applies ML algorithms and statistical analyses to predict firm growth. Finally, we conclude with a discussion. Importantly, with our vignette (and the associated online technical appendix in S1 Appendix), we hope to clearly illustrate the mechanics of our proposed methodology, including novel metrics and visualizations, so as to provide a menu of guidelines and techniques that can be readily used in future research irrespective of the ML algorithms used.

## Setting the scene

In advance, three remarks are worth making. Firstly, the methodology that we propose is intended to be used in multiple iterations, where each iteration gets us one step closer to the ultimate goal of better theorization, with an aim to increase the variance explained and to identify causal mechanisms (and prioritizing the latter). The research is cumulative over time, and the order of the five steps we propose here is not fixed. A single iteration tends not to reach the ultimate goal by using only one cycle of the methodology, and a cycle may be entered at another step of the sequence. This point is important because the ultimate promise cannot be delivered with a single vignette. The firm growth vignette illustrates how the current iteration provides concrete directions for the next iteration. On the one hand, the next iteration can improve the external validity of what we find in the current iteration by replicating the finding in other samples and examining boundary conditions. On the other hand, the next iteration can improve the internal validity by pinning down, finetuning and extending the causal mechanism that we suggest on the basis of the current iteration. And from one iteration to the next, we step by step reduce unexplained variance. Actually, this is how science does—or is supposed to—operate anyway. We return to these and related issues in the discussion section.

Secondly, complexity comes in many different forms and shapes. In this paper, we focus on the type where one outcome variable is linked in complex ways to a potentially large set of explanatory variables. Here, complexity emerges in two forms: non-linearities and interactions. In our vignette, the focus is on interactions, by way of illustration. In future work, other types of complexity can be explored. For instance, in the social sciences, a common type of complexity is that more than one outcome variable is involved, with these outcome variables featuring different types of reciprocal relationships. This leads to yet another important observation worth making in advance, relating to the meta-complexity of causality. As said, in the social sciences, good theory tends to be associated with causal explanations, or predictions. With observational data, the Holy Grail of the randomized controlled trial design is out of sight, although natural experiments and exogenous shocks might offer alternatives that come close (see, e.g., [22]). Hence, irrespective of the application of ML or classical methods, we cannot easily escape from imposing causality assumptions. For instance, supervised ML requires the up-front selection of a target or outcome variable, which implies the assumption that this target is caused by the other variables, or features, included in the analysis. We rather extensively return to the issue of causality below.

Thirdly, we should note that ML and classical methods have much in common. Both ML and classical methods share their foundation in the very idea of defining or learning a function ***y*** = *f*(***x***) that captures salient features of the data under study. But crucially, ML does not require the *a priori* specification of the ‘true’ data generation process, whilst classical methods do (for a careful comparison, see [9]). Given the latter, classical methods focus on $\hat{\beta }$—the values of the data generation process’ parameters estimated from the data, and the mapping of relationships between independent variables and the dependent. Instead, the algorithmic modeling culture of ML focuses on learning a function that operates on the features to predict the target in new, unseen data [23]. This focus on $\hat{y}$ rather than $\hat{\beta }$ translates into a higher tolerance for more complex functions of the predictors. Indeed, ML and classical methods share notable similarities, which are reflected in the metrics they produce, as will be seen below. Moreover, we note that classical methods are nested in the ML toolkit. A critical step in ML’s pipeline is to select the algorithm that ‘best’ fits with the data. Hence, if the data generation process under study largely satisfies all assumptions of the classical linear model, then this model would be selected.

## Background

### ML and understanding

ML is a very large and mature discipline at the interface of computer science and statistics. As the volume, velocity, veracity, and variety of data that society generates and collects are all increasing rapidly, the analysis of so-called Big Data transcends the cognitive capability of humans [24, 25]. Consequentially, there is a considerable and growing reliance on algorithms to structure, analyze, and model data. Although there is no consensus on the correct terminology for data-centric technology, the ‘machine learning’ label broadly refers to algorithms that ‘learn’ flexibly to optimize model performance criteria—such as the traditional and well-established *R*^2^, but particularly predictive accuracy—by evaluating generated (or ‘predicted’) output against observed but unseen (or ‘true’) data.

Alpaydin [26] offers the example of spoken speech to illustrate that people do some tasks, such as converting acoustic speech signals into written words, but cannot explain how they do this. Machine learning approaches this problem by collecting a large dataset of audio recordings and texts, and feeding all this into an algorithm. The algorithm may not necessarily transform the spoken speech into text in the way similar to what people do, but it can learn to do this transformation to produce output that makes sense to people. Machine learning algorithms can, in this fashion, construct a useful approximation that accounts for a surprisingly large part of the input data.

In this modern day and age, as computer science connects with data science and statistics, complex algorithms supported by high-performance computing platforms enable researchers to transform large piles of data into accurate predictions [27–30]. Yet, the predictions may not be causally interpretable or reproducible, as Judea Pearl [19] (p. 54) argued: “The dramatic success in machine learning has led to … increasing expectations for autonomous systems that exhibit human-level intelligence. These expectations have, however, met with fundamental obstacles that cut across many application areas. One such obstacle is adaptability, or robustness. Machine learning researchers have noted current systems lack the ability to recognize or react to new circumstances they have not been specifically programmed or trained for.” Moreover, a potential downside of machine learning techniques is that the process of transforming input into output, and hence the target system’s underlying causal mechanisms, can be rather incomprehensible [31]. This is a key area where, from a scholarly perspective, the research community is working hard to produce progress (we extensively return to this issue below).

Basically, this comes down to the critique that ML algorithms fail to live up to their promise of delivering understanding beyond pattern recognition and mechanistic prediction. To aid the causal interpretations and reproduce predictions across different contexts, Pearl and his co-authors advocate a systematic methodology for defining, estimating, and testing causal claims. The methodology is a convergence among certain philosophers, statisticians, and computer scientists over the last four decades on a process of theorizing and identifying causality (see [32, 33]). Numerous other scholars also made important contributions to the understanding of causality (e.g., [34, 35]). We build on this development, and review briefly the scientific methods for building theory with observational data, integrating ML techniques into a methodological cycle to combine their strengths with those of classic regression methods. Of course, we cannot but do this briefly; hence, we refer to the original sources for many detailed subtleties.

### Building theory with observational data

We take a theory, simply put, as a set of logical arguments, cemented together in a causal map that specifies the causes and causal mechanisms, in explaining or predicting an outcome. With observational data, we can build a model that predicts the probability of an outcome *Y* given certain determinants *D*: i.e., *P*(*Y*|*D*). To facilitate theory development, we rely on various scientific methods of reasoning, primarily known as deduction, induction, and abduction. We take our case of the firm growth puzzle to illustrate these three main methods of reasoning. To this threesome, we add two further steps, which we refer to as reduction and retroduction. We start with the workhorse of much hypothesis testing in the social sciences—deduction, defined as the inference of particular instances by reference to a general law or principle. Using *deduction* in the tradition of social sciences practice, we make a specific prediction from a hypothesis that is assumed to represent a general theory, and verify the extent to which the hypothesis is probable statistically. This is the classic probabilistic approach to quantitative empirical research in many social sciences. Indeed, in the firm growth literature, too, many examples abound of the deductive approach.

Suppose the outcome *Y* is a firm’s rate of growth, which is the change in the firm’s size over the time period [*t*, *t* + *τ*]. A classic hypothesis that has been assumed to be a general theory of firm growth states: Firm size follows a *random walk* [12]. A specific prediction from this hypothesis is deduced from a stochastic model of firm growth that describes the modification of firm size, over a given period of time, as the cumulative effect of a number of different shocks generated by the diverse accidents that affected the firm’s history in that period (see, among many, [36, 37]). One can write

$$g_{i}\left(t;\tau \right)=s_{i}\left(t+\tau \right)-s_{i}\left(t\right)=\sum_{j=1}^{G_{i}(t;\tau )}r_{j}\left(t\right),$$ (1)

where firm *i*’s rate of growth in the period [*t*, *t + τ*] is described as a sum of *G*_*i*_ (*t*; *τ*) ‘shocks,’ each one having a finite effect *r* on firm size. Gibrat [12] describes the growth process that he hypothesizes as a geometric Brownian motion. The growth rates associated with different nonoverlapping time periods are completely independent. When the number of shocks *G*_*i*_ (*t*; *τ*) increases, the rate of growth *g*_*i*_ (*t*; *τ*) tends, according to the Central Limit Theorem, toward a normal distribution. The unconditional distribution of *G*_*i*_ (*t*; *τ*), which is the unexpected shocks for a given firm, is binomial; so, in the limit of many diverse accidents such as the exploitation of technological novelties, the reaction to demand shocks, and the effects of managerial reorganizations, the probability density of growth rates has a bell-shaped Gaussian distribution. Manifestations of this classic random walk theory of firm growth are still alive and kicking (see, e.g., [17, 18]).

The consequence of the random walk model is thus that we can verify the extent to which the Gibrat hypothesis is probable statistically—namely, to what extent a normal distribution would fit the observed data on the growth rate distribution of business firms. The more variance in the observed data that can be explained by the model, the better the fit. To be clear, the fit is about a specific prediction deduced from the hypothesis. The hypothesis testing of the random walk model can only generate claims specific to the particular sample and the observed data. It cannot generalize from the particular (from past to future or from observed to unobserved). The most general claim is a set of stylized facts about the stochastic trends of growth rates and the unpredictable nature of shocks that makes it difficult to predict what a firm’s size will be at any time in the future, *t + τ*. Based on the stylized facts that are consistent with Gibrat’s hypothesis, firm growth appears to be an idiosyncratic and fundamentally random process, as stated by Geroski [37].

The Gibrat hypothesis focuses on stochastic shocks. As a response, competing hypotheses that focus on management have emerged in the literature that emphasize a wide variety of managerial sources of non-randomness. For instance, Stanley et al. [38] propose a model of firm growth that relies on a *technology of management*. Their management model of firm growth was developed based on a Simonian methodology, whereby facts are first pursued through empirical investigations and then theories are formulated as attempts to explain the facts that emerge. New stylized facts have emerged, showing, rather than the bell-shaped Gaussian, that a tent-shaped distribution derived from a Laplace (symmetric exponential) function gives a better fit with the observed data. Using a sample of all publicly traded US manufacturing companies between 1975 and 1991 from the Compustat database, Stanley et al. [38] find that the random walk model fails to explain the observed probability densities of the growth rates. The growth rate distribution is not Gaussian, as expected from the Gibrat hypothesis, but rather is exponential. Amaral et al. [39] also find a tent-shaped probability density to be a better fit with the observed data using a sample of all publicly traded US manufacturing companies between 1974 and 1993 (see also [40]). And so do Bottazzi and Secchi [41], using a sample of Italian manufacturing industry that is analyzed sector by sector, and Bottazzi et al. [42], analyzing a sample of firms in the worldwide pharmaceutical industry.

The technology of management theory of firm growth is just one example out of many managerial theories of firm growth. Another one is the management theory of gazelles (see, e.g., [43]), which emphasizes the role of management’s strategic choices. What suffices to observe in the context of the current paper’s message and vignette is that in light of the focus on management and the emerging stylized facts, we add a first step to our cycle, which we label *reduction*—selecting potentially influential explanatory variables for an outcome variable based on researcher observation and literature review—to select the explanatory variables of *Y*. In the context of our vignette, the type of variables covers the characteristics of managers, which we call *Z*. Another type covers managerial decisions, which we refer to as *X*. *Z* and *X* are the key explanatory variables in a model of firm growth that focuses on management. Below, we will provide more details about *Z* and *X* in the vignette.

Next, we turn to induction, defined as deriving general principles from a body of observations. In our context, we use *induction* in the sense of exploring patterns in the data to infer something about the future course of events, making a specific prediction by transferring a property or regularity from the past to the future, or from one sample to another in the narrow Humean sense. Simpson’s paradox [44], which refers to the existence of data in which a statistical association that holds for an entire population is reversed in every subpopulation (see [45]), reminds us of David Hume’s [46] philosophical problem of induction. How do we know that the future will be like the past? Through an ML lens, how certain is our inference about the *Z*-*Y* association that is observed in the training sample, if the association is confirmed in the validation sample? And how certain is our inference about the *X*-*Y* association that is observed in the training sample, if the association is confirmed in the validation sample? The methodology advocated by Pearl et al. [47] provides a guidance for building a model in which *Z* and *X* are causes of *Y*. To build such a model, we need what we refer to as *retroduction*, which involves developing hypotheses to infer something about the unobserved causes of the observed events.

Using *retroduction*, we hypothesize what causes *Y* (cf. [48, 49]). The method of retroduction was advocated by Hanson [50] (p. 85–88), who followed Peirce [51] and influenced Simon [52] (p. 339, 443, & 456). Retroduction is often referred to as ‘the official Peirce abduction schema’, factual abduction, or what Peirce [51] called hypothesis, a form of reasoning that extracts from data an explanation that accounts for particular observations [10] (p. 206). This method derives generalizations from data by observing raw data, and concluding that the observed data can be described adequately by a specific theoretical model. In the model, causes and causal mechanisms are hypothesized. Retroducing causes and causal mechanisms “is of central importance for manipulating the course of events, that is, adapting the course of events to our wishes” [10] (p. 202). The ability to manipulate the course of events relies on a causal inference about a property or regularity that transfers from the past to the future, or from one sample to another.

**Fig 1:**
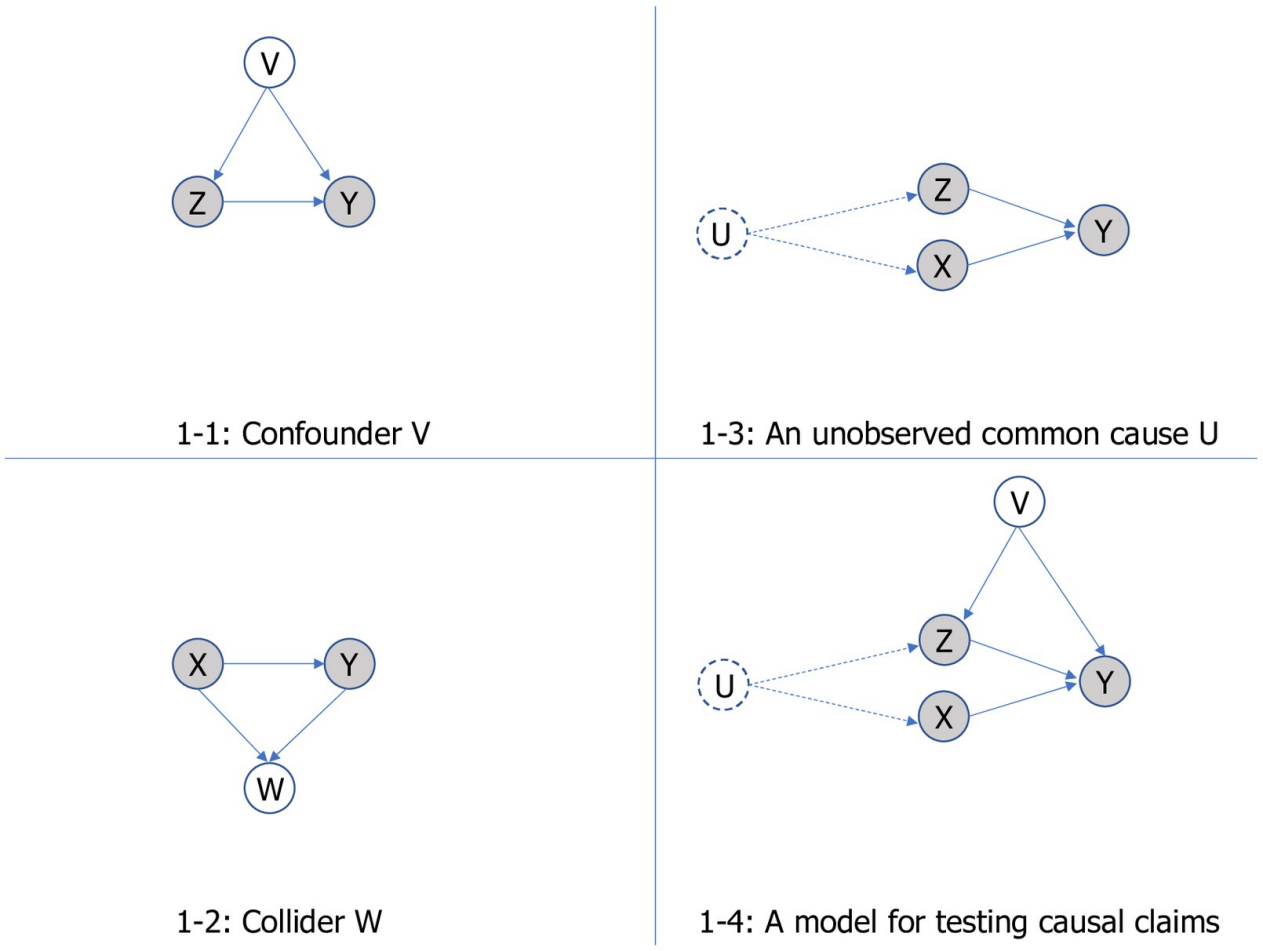
Building a model for predicting the rate of firm growth. Notes: Y = outcome or target variable (firm growth in the vignette); Z = personal trait variables (egocentrism in the vignette); X = managerial decision variables (customer intimacy, operational excellence, and product leadership in the vignette; and V = control variables (financial and size measures in our vignette).

**Fig 2:**
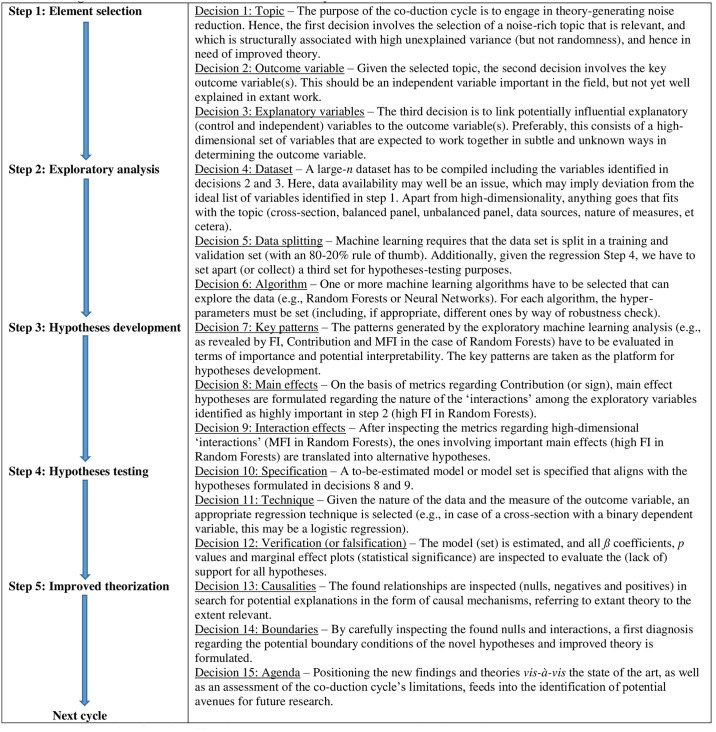
A flowchart of the co-duction cycle. Note: Illustrated for the case of Random Forests (for regression), with FI = Feature Importance and MFI = Most Frequent Interactions.

As an illustration of *retroduction*, we build a model in which *Z* and *X* are causes of *Y*. Following Pearl et al.’s [47] methodology for defining, estimating, and testing causal claims, our model has three components. Fig 1 illustrates how modeling and causality are related. In each component, we hypothesize the causes and the causal mechanisms. The first component hypothesizes a confounder *V*: *V* is a common cause of *Z* and *Y* (see the graphical model in Fig 1-1). A prediction about the causal effect of *Z* on *Y* is biased when confounder *V* is not included in a model [47] (p. 62). The second component hypothesizes a collider *W*: *W* is a common effect of *X* and *Y* (see the graphical model in Fig 1-2). A prediction about the causal effect of *X* on *Y* is biased when collider *W* is included in a model [47] (p. 48, 63).

Suppose doing *X* and growing fast are both associated with the probability of receiving venture capital funding. Including a variable that indicates whether the firm receives venture capital funding makes the prediction of *X*→*Y* biased. Controlling for *W* is to compute the effect of *X* on *Y* for each value of *W* separately. Doing so will not yield the correct effect; in effect, it might even give the wrong effect for each value of *W*. Controlling for whether the firm receives venture capital funding would bias the estimated effect of *X* because doing so filters the sample into two groups. One group receives funding, whereas the other does not. *W* generates a dependence between *Z* and *Y*. That is, the group-specific effect is a conditional dependence that is created by including *W* as a control variable. When controlling for *W*, the estimation of *X*→*Y* is biased.

**Fig 3:**
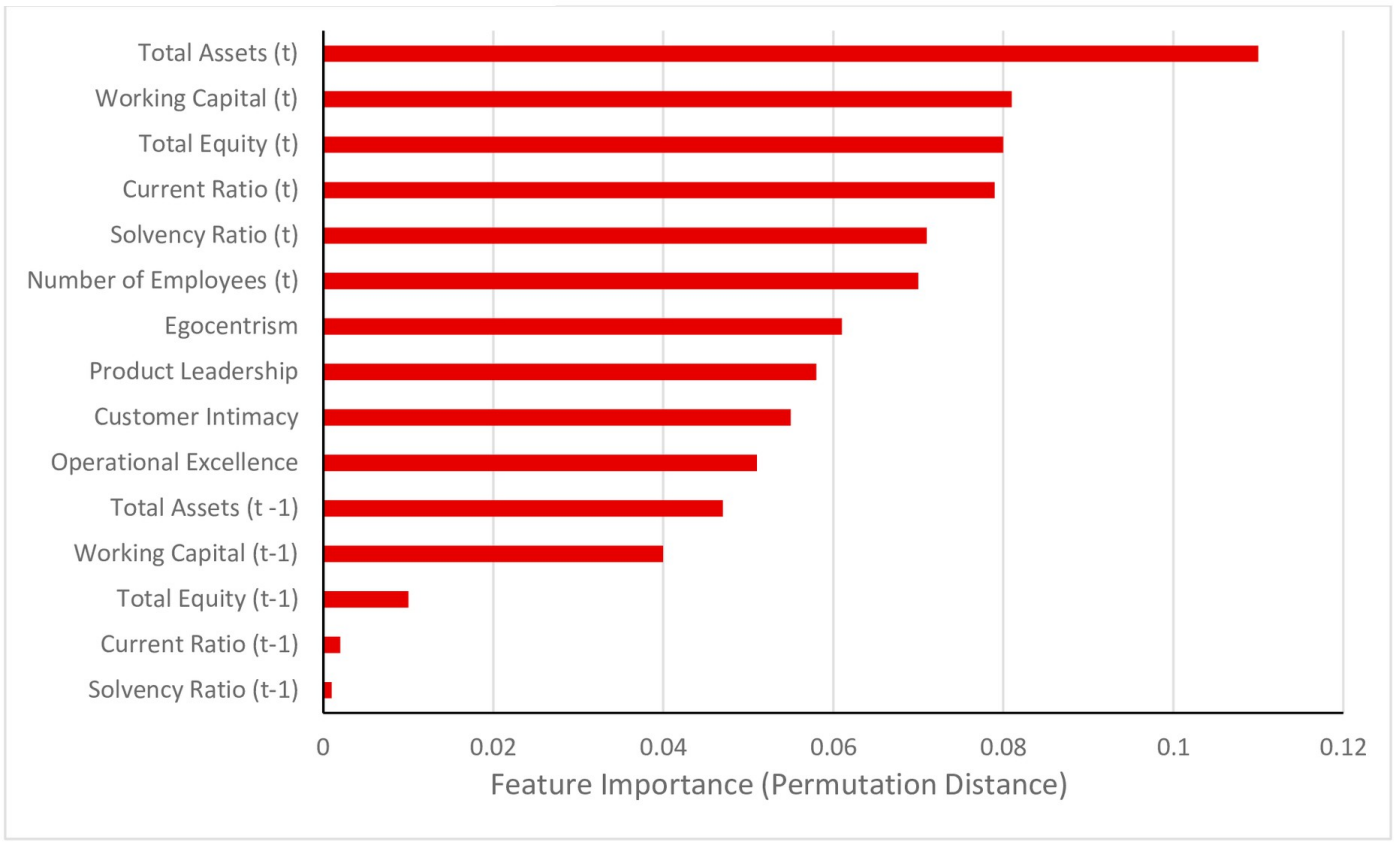
Feature importance.

**Fig 4:**
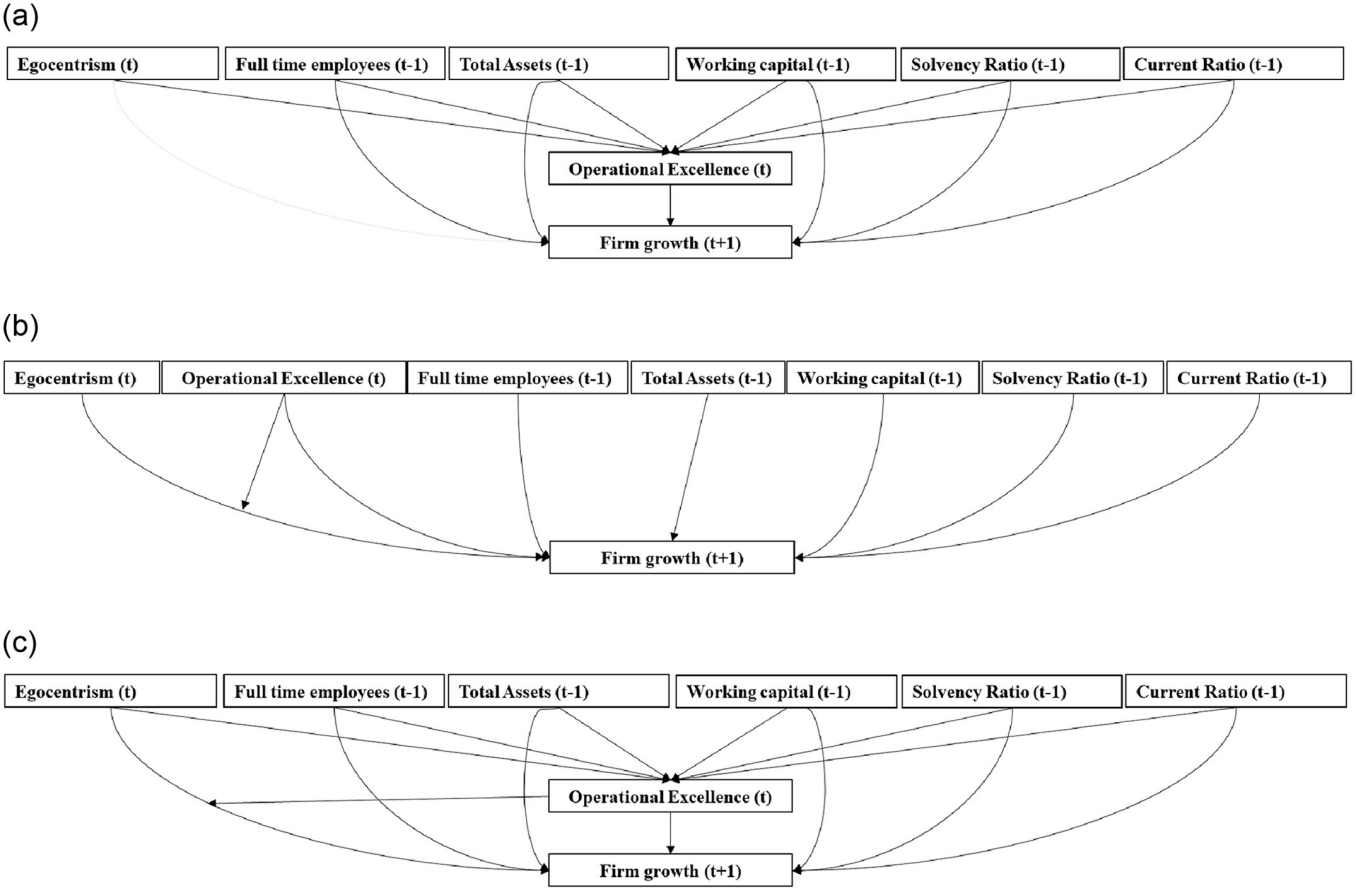
**a:** Visualisation of Hypothesis 2. (The effect of Egocentrism on firm growth fully runs through the positive association of Egocentrism with Operational Excellence.) **b:** Note: For the sake of completeness, the influential control (*V*) variables are visualized as well. (The effect of Egocentrism on firm growth is positively moderated by Operational Excellence.) **c:** Visualisation of Hypothesis 1 through 5 in a nested overall model. Note: For the sake of completeness, the influential control (*V*) variables are visualized as well.

The third component hypothesizes an unobserved common cause of *Z* and *X*: *U* is an unobserved variable that causes *Z* and *X* (see the graphical model in Fig 1-3). Suppose *U* is unmeasured or not recorded. If *X*, which is not a descendant of *Z*, blocks the path *Z*→*U*→*X*→*Y*, then including *X* in modeling *Z*→*Y* will give us the causal effect of *Z* on *Y* [47] (p. 62). Putting these components together, we arrive at a model that hypothesizes why firms with characteristics *Z* grow faster, and why firms that do *X* grow faster (see the graphical model in Fig 1-4). For *Z*→*Y* and *X*→*Y* to be eligible as causal claims as presented in the graphical model, confounder *V* has to be included in the model as a control variable, collider *W* cannot be included in the model, and *X* cannot be a descendant of *Z*.

We further illustrate *deduction* with the model. If we assume that the model as presented in Fig 1 is true, we then deduce specific predictions that are the consequences of the hypothesized causes (nodes in the graphical model) and causal mechanisms (paths in the graphical model). The first set of consequences pertains to the conditional independence between variables. If *V* is a common cause of *Z* and *Y*, and there is only one path between *Z* and *Y*, then *Z* and *Y* are independent conditional on *V*. If *W* is a common effect of *X* and *Y*, *W* generates a dependence between *Z* and *Y*. That is, *X* and *Y* are unconditionally independent, but they are dependent conditional on *W*. The conditional independence can be tested empirically using regression techniques.

The second set of consequences pertains to the model’s fit with the observed data. The fit can be tested empirically using regression techniques as well. The graphical model corresponds to a system of equations, guiding model specification to include *V* as a control variable when *Z* and *X* are the explanatory variables. *W* cannot be a control variable. The third set of consequences pertains to the interaction between *Z* and *X* as a cause of *Y*. If *X* is a moderator that modifies the degree to which *Z* affects *Y*, the *Z*-*Y* conditional probability is different at different values of *X*. Namely, *X*-specific effect of *Z*→*Y* is the consequence of having *X* that blocks the path *Z*→*U*→*X*→*Y*. The interaction between *Z* and *X* is a specific prediction that can be tested empirically using regression techniques. For instance, the effect of *Z* on *Y* is stronger for firms that do *X*. We will provide more details about the interaction between *Z* and *X* in the vignette on firm growth. Note that here ‘interaction’ is clearly defined as moderation (we return to this issue below).

Lastly, we make use of *abduction*, which is defined as inference to the best explanation. We use abduction to improve theorization by producing novel theoretical models that increase predictive power. Specifically, we seek to come up with a model that fits well with the observed empirical phenomena by explicitly identifying plausible causal mechanisms. A model’s fit with the observed empirical phenomena highlights increased predictive power. Predictive power as a criterion for evaluating an inference is different from the criterion used for inference to the best explanation (often referred to as IBE). Ockham’s razor is a broadly accepted maxim for evaluating an IBE: An explanation of observed phenomena should postulate as few unobservable or new entities or properties as possible [10] (p. 219). Yet, where is the borderline between ‘reasonably many’ and ‘too many’ entities postulated for the purpose of explanation? If a phenomenon is novel and poorly understood, then one’s best available explanation is usually a pure speculation. Purely speculative abduction are pseudo-explanations that have no predictive power at all [10] (p. 220). Consistent with Schurz [10], we declare that increased predictive power is the objective of building a better model. The predictive power increases when the model has strong determinants.

## A cycle of co-duction steps

We are now ready to synergistically and sequentially cement together the above three methods of reasoning (abduction, deduction, and induction) and both extra steps (reduction and retroduction) into what we refer to as a ‘co-duction’ cycle aimed at developing better theories, by benefiting from the complementary strengths of modern ML and classic regression techniques. As will become clear below, across the cycle, each step in the cycle gives prominence to a different aspect of the route toward theory formation introduced above in the following order: (1) reduction; (2) induction; (3) retroduction; (4); deduction; and (5) abduction. We introduce the neologism ‘co-duction’ to characterize our novel methodology, as new theory is developed by *co*mbining the five steps that all end with *duction*. The cycle is summarized in Table 1 and Fig 2.

**Table 1:** The five-step co-duction cycle.

Step	1: Element selection	2: Exploratory analysis	3: Hypotheses development	4: Hypotheses testing	5: Improved theorization
**Methods**	Reduction	Induction	Retroduction	Deduction	Abduction
**Activity**	Observing	Identifying	Recognizing	Quantifying	Understanding
**Outcome**	Inspiration	Prediction	Interpretation	Estimation	Attribution
**Agent**	Human	Machine	Human	Machine	Human
**Action**	Carving	Exploring	Reasoning	Regressing	Thinking
**Decision**	1. Topic 2. Outcome variable 3. Explanatory variables	4. Dataset 5. Data splitting 6. Algorithm	7. Key patterns 8. Main effects 9. Interaction effects	10. Specification 11. Technique 12. Verification (or falsification)	13. Causalities 14. Boundaries 15. Agenda
**Criteria**	1. Research gap 2. Relevance 3. Noise	1. FI 2. Contribution 3. MFI	1. Importance 2. Interpretability 3. Plausibility	1. *ß* coefficient 2. *p* value 3. R square	1. Inference 2. Narrative 3. Novelty
**Elucidation**	Based on the literature and observation, a research gap is identified that is both relevant and associated with much noise (unexplained variance), but non-dominant randomness. Inspired by the state of the art and further thinking, a reduction exercise is performed to carve out the key variables that may, in yet unknown and potentially complex ways, interact in determining the outcome.	Machine learning is applied to inductively explore the data in order to identify prominent patterns among the variables in terms of Feature Importance (FI), direction of Contribution (- or +) and high-dimensional interaction linkages (MFI), such that the prediction error is minimized (but balanced against overfitting).	Inspection of the identified patterns by the machine learning algorithm feeds into retroductive reasoning in search for plausible and alternative interpretations that can be formulated in the form of important hypotheses that can back up the inductively identified patterns theoretically.	The formulated hypotheses are deductively tested using traditional regression techniques on a new dataset, generating parametric estimates of all modeled *ß* coefficients, being associated with *p* values, quantified effect sizes and *R*^*2*^, leading to verification or falsification of the proposed hypotheses, and identification of the nature of ‘interactions’.	The relationships revealed by the regression are linked to a causal logic in an attempt to abductively develop a convincing narrative of inference that provides novel understanding regarding attribution mechanisms, explaining the found results by specifying the underlying causal mechanisms.

Note: Illustrated for the case of Random Forests (for regression), with FI = Feature Importance and MFI = Most Frequent Interactions.

What we do in our co-duction cycle is linking the aforementioned steps in the theory formation pipeline to practical activities, actions, and decisions conducted by researchers to produce specific outcomes on the basis of certain criteria. Moreover, and critical to our methodology, we explain how modern ML and classic regression techniques could be combined, in different steps of the co-duction cycle. Our co-duction cycle is composed of five distinctive steps, each associated with specific methods, activities, outcomes, agents, actions, criteria, decisions, and techniques. Specifically, we provide a five-step and easy-to-apply roadmap, in which non-parametric quantitative pattern-identifying induction through machine learning feeds into traditional deductive parametric hypotheses-testing statistics, ending with abductive theorization. Below, we briefly introduce the essence of each step. In the vignette, we further illustrate our five-step cycle, including the associated fifteen decision points.

*Step 1* relates to *element selection*. After selecting the research topic, this first step involves *reduction* as a method for selecting potentially influential explanatory variables for an outcome target based on researcher observation and literature review. The purpose is to identify and select the elements (i.e., the potentially relevant theoretical building blocks of a general framework) to be utilized in the subsequent step for empirical analysis. This step is based on human judgement, for which the researcher is the key agent. Based on the literature and observation, a research gap is identified that is both relevant and associated with considerable noise (i.e., unexplained variance). Inspired by the state of the art and further thinking, a reduction exercise is performed to carve out the key variables that may, in yet unknown and potentially complex ways, work together in determining the outcome.

*Step 2* is an *exploratory analysis*. This second step involves *induction* as a method for exploring patterns in the data to infer something about the future course of events. It is a comprehensive empirical analysis assisted by automated ML techniques and standard sample-splitting practices (creating a training sample and a validation sample for the ML analysis, plus a test sample for the hypotheses-testing Step 4). Splitting the total data into three separate datasets is essential to avoid any information leakage.

The logical connections among the elements from Step 1 are explored in Step 2 using quantitative pattern-identifying ML techniques. In this step, for which the agent is a machine, the key variables explaining the outcome of interest are identified algorithmically according to the order of their importance, including the “interactions” among the key variables. The inductive data exploration aims to reveal prominent patterns among the variables in terms of Feature Importance (FI), direction of Contribution (- or +) and high-dimensional Most Frequent Interaction linkages (MFI), in ways that model fit is maximized or, rather, optimized (that is, the prediction error is minimized while balancing under and overfitting). We note that, in ML, ‘interactions’ are a broader category than moderation in classical regression as it is used in the social sciences. We return to this issue below.

*Step 3* focuses on *hypotheses development*. This third step involves *retroduction* to develop hypotheses to infer something about the unobserved causes of the observed events. Based on the patterns learned in the Step 2’s exploratory analysis, specific hypotheses or propositions are developed. As in Step 1, this requires human judgment, for which the researcher is the key agent, because the patterns explored with ML are subject to human interpretation. The purpose of Step 3 is to carefully reflect upon the order of Feature Importance (suggesting possible main effects) and the Most Frequent Interactions (pointing to possible mediation or moderating effects). Inspection of the patterns feeds into retroduction in search for plausible interpretations that can be formulated in the form of alternative hypotheses that are consistent theoretically with the inductively identified patterns. We note that, in the context of our illustrative application of the Random Forest ML algorithm, the order of Feature Importance and the Most Frequent Interactions are the ML metrics we take on board. Of course, there is much more out there, which is evolving quickly (see, e.g., [53]).

*Step 4* is *hypotheses testing*. This fourth step involves *deduction* as a method for testing hypotheses by making a specific prediction from a hypothesis that is assumed to be part of a general theory, and verifying the extent to which the hypothesis is probable statistically. The hypotheses are tested by running appropriate multivariate regression analyses on a part of the dataset unseen in the ML step 2. In so doing, Step 2’s non-parametric patterns explored with ML are transformed into parametric estimations we are so familiar with in the social sciences, permitting the usual interpretations of findings in terms of statistical significance, confidence interval, and effect size. Hence, as in Step 2, the machine is the key agent. The formulated alternative hypotheses are deductively tested using traditional regression techniques, generating parametric estimates of *ß* coefficients, being associated with *p* values, quantified effect sizes and *R*^*2*^ (or similar metrics of model fit), leading to verification or falsification of the proposed hypotheses. As we noted earlier, the use of separate datasets for hypothesis generation and hypothesis testing is essential. Failing to adopt the standard sample-splitting practices would violate the assumptions of deductive hypothesis testing (see [54]) and lead to a questionable practice resembling hypothesizing after the results are known (HARKing). We return to this issue below.

*Step 5* concludes the cycle, leading to improved *theorization*. This fifth step involves *abduction* as a method of reasoning for improving theorization by producing novel causal maps to increase our understanding of complex social reality. The estimations from Step 4 are carefully interpreted in Step 5 so as to theorize plausible underlying causal mechanisms and produce novel contributions to theory development. This is the final step of the co-ductive five-step roadmap, kickstarting the next cycle. The interpretations of findings by the researcher as the key agent are the bread and butter of the social sciences’ dominant research practice. The relationships revealed by the regression are linked to a causal logic in an attempt to abductively develop a convincing narrative of inference that explains the findings by specifying the underlying causal mechanisms.

To summarize, this five-step cyclical co-ductive roadmap combines a mix of techniques associated with different methods of reasoning. Specifically, after a reduction step, ML offers inductive non-parametric input for subsequent retroductive theorizing that provides the input for deductive parametric multivariate regression analyses, leading to abductive theorization. ML offers a quantitative method of induction that can be combined with traditional deductive techniques, using the output of the first to identify input for the second. Together, these steps produce the input for novel theorization, feeding into abductive theorization. Importantly, as we will illustrate below, alternative or competing hypotheses are developed and tested along the way, implying an advancement beyond the heavily criticized (but still highly dominant) practice of null hypothesis statistical testing, where the primary focus is on testing a no-theory null (e.g., [55, 56]).

As mentioned earlier, ML algorithms are designed to optimize predictive accuracy. Step 2 is an automated learning exercise that produces high model fit based on ensembles of predictors that ‘interact’ non-linearly in complex and high-dimensional ways. Subsequently, regression in Step 4 is designed to examine the precise nature of the key ‘interactions’, examining different alternative causal mechanisms (e.g., mediation versus moderation). In the vignette that we will illustrate in the next section, we show how our Random Forest Analysis produces a model with a high *R*^2^, which is due to a set of determinants working together to produce a (very) good prediction. This then inspires the selection of clearly specified main, mediation, and moderation hypotheses to be deductively examined in the subsequent regression that facilitates the screening for important determinants and the nature of their relationships through the classic effect size–*p* value metrics.

## A vignette

We demonstrate an application of our five-step co-duction research methodology with the classic puzzle of firm growth. Throughout the vignette, we italicize the decisions that are made as part of the cycle and refer to their corresponding step in Table 1. In advance, we would like to reiterate two remarks that we made in the Introduction. First, with our vignette, we can only illustrate the workings of all steps in the full cycle without any claim that this is sufficient to reach the ultimate goal of developing theory associated with an impressive *R*^2^, to stick to a traditional metric. For that, multiple—often many—iterations are needed. Also, as we will further discuss in the Conclusion, not each and every single study is required to progress through all five steps of the full co-duction cycle, and does not have to do so in the same order or in the same way. Second, by selecting firm growth as our target variable, required to conduct supervised ML, we impose a causal map onto our analysis from the very beginning. However, as we explain further down the line, we use our co-duction cycle to, step by step, come closer to a theory specifying the precise nature of the underlying causal mechanisms.

Regarding data collection, we scraped “About Us”-pages of websites of Belgian medium and small-sized enterprises (SMEs) in 2016. After discarding empty pages, we ended up with 8,163 SMEs with valid pages. We then collected panel data on demographic, financial, and size indicators for those firms for the years 2016 and 2017. We used this dataset for the inductive machine learning Step 2 of our co-duction cycle, with firm growth in 2017 as the target. We divided this ML part of our data in a training (learning) and a test (validation) set with an 80% − 20% split. The training set was used for hyper-parameter tuning and model training. The models were evaluated against the test set to get a sense of how well they would generalize to new, unseen data. After having selected the best hyper-parameters, we retrained the Random Forest on the complete dataset and then computed the explanatory outputs included in the paper. For the deductive traditional regression Step 4 of our co-duction cycle, we re-scraped the “About Us”-pages in 2017 and added data on demographic, financial, and size indicators for 2018, taking firm growth in 2018 as the dependent variable. The regression analyses and structural equation models were done on this new dataset that was not used in the machine learning part of the analysis, and which is thus unseen by the Random Forest.

So, our data are, effectively, sliced in four parts. The first slice was used in an earlier study [21]. The second and third slice are the current study’s ML learning and validation data set. The difference with the first slice is that extra features have been added by web scraping and text analyzing Internet pages of 8,163 firms. Hence, as a result, the current paper’s sample size is much lower than that in the earlier study (with an *n* of 168,055). These training and validation data are analyzed in Step 2 of our co-duction cycle. The fourth slice is constructed by moving our time window with one year for our 8,163 observations, from 2016–2017 to 2017–2018. For this, we re-scraped the web pages in 2017 (*vis-à-vis* 2016 in Step 2) to produce new measures of our text-analyzed features for the deductive traditional regression analyses in Step 4. In all, this means that we avoided any data leakage from the inductive Step 2 (and within Step 2) to the deductive Step 4, in line with the ML’s process pipeline best practices.

### Step 1: Element selection—Reduction

As mentioned earlier, the empirical findings on the rate of firm growth show much unexplained variance. This may be due to ‘true’ randomness, but further work is needed to explore potential non-random drivers of firm growth, particularly those suggested by the managerial perspective. Thus, firm growth is a *topic* (D.1) worthy of further investigation. The rate of firm growth, the *outcome variable* (D.2), referred to as Firm Growth in our analyses, is defined as:

$${Growth}_{it}=\left(\frac{A_{it}}{A_{it-1}}\right)\cdot 100,$$ (2)

where *A*_*it*_ is the total assets in Euros of firm *i* at time *t*.

The literature suggests a large variety of factors that could contribute to firm growth. In the managerial theory of firm growth, characteristics of managers (*Z* in Fig 1) and strategies (*X*) take center stage. We start with a set of *explanatory variables* (D.3) including 16 potential predictors that van Witteloostuijn and Kolkman [21] compiled from the literature on firm growth. Indeed, this set of 16 potential predictors are all financial or size measures identified in prior work as potentially important (cf. [14] Coad, 2009). In the context of our vignette, and with reference to Fig 1, we include these *V* variables as controls. Theoretically, our focus is on managerial perspectives on firm growth. We augment the set with managers’ personality trait of egocentrism/narcissism [57] as suggested by behavioral strategy literature [58]. Personality trait is an important *Z* variable relating to characteristics of managers in our theoretical model (see Fig 1). We further augment the set with managerial decisions as examples of *X* variables in our theoretical model. The managerial decisions are suggested by Treacy and Wiersema’s [59] classic threesome of value strategies: (1) operational excellence; (2) product leadership; and (3) customer intimacy.

### Step 2: Exploratory analysis—Induction

Our *dataset* (D.4) is van Witteloostuijn and Kolkman’s [21] baseline database with *V* control variables, enriched with time lags and augmented with *Z* and *X* variables. To the baseline dataset, we added a one-year time lag for balance sheet total (or total assets), total equity, working capital, current ratio, solvency ratio, and number of employees. The legal person and sector of a firm are categorical variables, which we transformed into a series of binary dummies. This resulted in a total of 113 independent ‘features’ or predictors.

Our central *Z* and *X* variables are constructed by processing natural language data that we collect from the websites of 8,163 Belgian and Dutch small and medium sized enterprises (SMEs). All French-language SMEs were removed to avoid any translation issues. If the Dutch word is too distant from its English counterpart to reveal its meaning to readers not fluent in Dutch, we added a translation in English. Table 2 lists the groups of words that were used to generate the *Z* and *X* variables. In addition, we apply a topic modeling algorithm to process the massive amount of rich content. Topic modeling algorithms are a class of unsupervised ML techniques that identifies groups of words that represent a shared topic across a corpus of texts (Shu et al., 2009). Specifically, we apply a topic modeling algorithm known as Latent Dirichlet Allocation (LDA; see [60]). Examples of applications in the business and policy field are Kaplan and Vakili [61] and Haans and van Witteloostuijn [62].

**Table 2:** Groups of words for our Z and X variables.

	Topic	Group of words
**Z variable: managers’ characteristics**	Egocentricity	’ik’ (‘I’), ’mij’ (‘me’), ’mijn’ (‘mine’), ’mijzelf’ (‘myself’)]
**X variables: managerial decisions**	Product leadership	’technologie’, ’innovatie’, ’vernieuwing’ (‘renewal’)
	Customer intimacy	’service’, ’maatwerk’ (‘tailor-made’), ’kwaliteit’ (‘quality’)
	Operational excellence	’korting’ (‘discount), ’uitverkoop’ (‘sales’), ’sale’

For pattern exploration, we use a model-fit optimizing algorithm called Random Forest Analysis (RFA), following van Witteloostijn and Kolkman [21]. RFA is a frequently used automated *algorithm* (*D*.*6*) for classification and regression in ML. RFA generates: (i) a rank order of variables based on their explanatory/predictive power [i.e., in terms of Feature Importances (FI)]; (ii) the key variables that ‘interact’ with the focal variable [i.e., Most Frequent Interactions (MFI)]; and (iii) a non-parametric Contribution metric. Strictly speaking, following the standard ML pipeline would require separate analyses aimed at finding the ‘optimal’ algorithm–that is, the one that performs ‘best’ on the data at hand. In the spirit of building a cumulative body of evidence and illustrating our main co-duction argument, however, we choose RFA as our model-fit optimizing algorithm because it was used by van Witteloostuijn and Kolkman [21].

We apply the RFA without any restriction regarding functional specifications or higher-order ‘interactions’. As briefly explained below, we minimize model overfitting by carefully pre-specifying hyper-parameters, and by running analyses with both a training sample and a validation sample.

We make three choices, as we follow the estimation procedure suggested by Putka et al. [63]. First, we choose an 80–20 ratio when randomly splitting the data (*D*.*5*) to create a training (80% of the data) sample and a validation (20% of the data) sample. Second, we choose 30 folds in the k-fold cross-validation [64] to determine the hyper-parameters. Specifically, we set an input list of hyper-parameters. Then we conduct a random search [65] to identify hyper-parameters with the highest *R*^2^. We select hyper-parameters following the 1-SE rule to “choose the simplest model whose accuracy is comparable with the best model” [66] (p. 11). For models with less than 100 degrees of freedom with respect to the hyper-parameters, we take the maximum degree of freedom. The third choice we make is to fit the RFA with the best-performing hyper-parameters to the training sample using 30-fold cross-validation. This results in an impressive maximum *R*^2^ of 0.63 on the validation sample. An *R*^2^ of 0.63 is extremely high for any study in the social sciences, and certainly so in the firm growth literature. Importantly, adding the LDA-generated *Z* and *X* variables increases the *R*^2^ from 0.48 to 0.63. We note that, as a point of comparison, the R^2^ is 0.23 in [21]. The higher R^2^ in the current paper is probably due to two differences. First, our sub-sample of 8,163 firms is biased toward larger firms, and is associated with much higher variability. Second, only demographic, financial and size variables were included in their analysis.

Fig 3 reports Feature Importance (FI), revealing the most important variables in explaining the Firm Growth outcome. For each of the features listed in Fig 3, we inspect the decision trees that are generated by the RFA, and count what other features they ‘interacted’ with in the decision trees that make up the RFA. We do so for the cases with an absolute percentage error smaller than 10% as reported in Table 3. Table 3 lists the top five Most Frequent Interactions (MFIs) per feature, as well as the Contribution metric of each feature to the explanation of prediction of the outcome variable. The Contribution metric is (very) roughly equivalent to the *β* coefficient in a regression model [67], but should be interpreted with caution as the RFA is a non-parametric technique. It would be a mistake to take this as evidence that there is a linear relationship between the features and the outcome variable. The Contribution metric does not provide definitive insights regarding effect size. While the *Z* and *X* variables feature in Fig 3, the LDA-generated factors do not appear amongst the highest-ranked features, appearing on position 7 to 10.

**Table 3:** Feature contributions and top-three feature interactions (MFIs).

#	Variable	MFI	Contribution
1	Total Assets (*t*)	3,11,13	0.09
2	Working Capital (*t*)	3,13,11	-0.02
3	Total Equity (*t*)	1,11,13	0.77
4	Current Ratio (*t*)	3,13,11	-0.07
5	Solvency Ratio (*t*)	3,13,15	-0.02
6	Number of Employees (*t*)	3,1,11	-0.03
7	Egocentrism	3,4,11	0.02
8	Product Leadership	1,4,14	0.02
9	Customer Intimacy	3,1,11	0.03
10	Operational Excellence	3,1,11	-0.04
11	Total Assets (*t* -1)	3,13,1	0.15
12	Working Capital (*t*-1)	3,11,13	-0.02
13	Total Equity (*t*-1)	3,11,1	0.13
14	Current Ratio (*t*-1)	3,5,13	-0.04
15	Solvency Ratio (*t*-1)	3,1,11	0.00

An important remark is in place regarding the meaning of ‘interaction’ in the context of RFA (or any other ML technique, for that matter). In the social sciences, interaction has the very specific meaning of moderation, as reflected in product terms included in traditional probabilistic regression analyses. In RFA, the meaning is much broader. In the ML literature, ‘interaction’ is used to refer to a more general class of association between independent (or predictor) and/or dependent (or outcome) variables. This meaning of the concept is grounded in statistics. The *Oxford Dictionary of Statistical Terms* entry on interaction (2003, p. 203) suggests that “While sometimes used in the broad sense of effects not operating separately, in statistical discussions it is typically restricted to effects that do not act additively on some response variable.” This implies a broader interpretation of ‘interaction’, which is confirmed in the next lines where three types of interactions are listed: “Positive interaction between two explanatory variables corresponds to the biological notion of **synergism**. When there is an interaction between an explanatory feature and a background variable the latter may be called an **effect modifier**. (‥) Interactions involving effect reversals may be called **qualitative**.” None of this pertains to the direction of causality, but implies that ‘interaction’ can encompass different types of association.

Somewhat confusingly, Breiman [68], who laid the foundations for RFA, define ‘interaction’ as variables m and k interact if a split on one variable, say m, in a tree makes a split on k either systematically less possible or more possible. This emic definition seems to revolve entirely around how associations between variables work in a Random Forest, and not around how such associations should be typified. Breiman does suggest that if variable m1 is correlated with variable m2 then a split on m1 will decrease the probability of a nearby split on m2. In line with this ambiguity, Cutler et al. [69] mention complex interactions, Hayes et al. [70] refer to moderators, and Zhao and Hastie [71] talk about mediation in the context of Random Forests, none of these studies does offer a clear definition of any of these concepts. In lieu of any definitive statements on the nature of associations modeled by Random Forests, we conclude that in the absence of any one clear interpretation of the ‘feature interactions’ in RFA outputs in the context of causal models, we have to turn to (causal) theory and traditional regression (see below) to further explore and examine the nature of what is referred to as ‘interaction’. Specifically, we need other steps in the co-duction cycle to reveal whether the RFA-identified MFI variables involve (full or partial) mediation or moderation, or a combination thereof. Here, the first step is to engage in retroduction.

### Step 3: Hypotheses development—Retroduction

It is important to briefly touch upon the relative weight we attributed to various RFA visualizations and other outputs. A full discussion of each of these techniques can be found in the Online Technical Appendices in S1 Appendix, but we offer our most important considerations here. The Feature Importances, Contributions, Most Frequent Interactions, one-way partial dependence plots, and two-way partial dependence plots are different ways to explore the model learned by the RFA and identify *key patterns* (D.*7*). The interpretation of these outputs and visualizations should be done with care, as each has its own purpose, but each is also subject to one or more weaknesses. Since RFA is used in Step 2 as part of the co-ductive methodology, we put more weight on techniques that employ the entire dataset and utilize the full Random Forest. That is, more weight is assigned to techniques that compute Feature Importance and Contribution values using the entire dataset and covering all trees in the Random Forest. The MFIs are computed for a subset of data for which the Random Forest has an absolute percentage error smaller than 10%. Since this is a subset of data, we rank this output just below Feature Importance and Contribution, but above the partial dependence plots. The one-way and two-way partial dependence plots are computed for a random subset of 900 and 20 data points, respectively. As such, we treat these visualizations as merely indicative.

We now turn our attention to the outputs and visualizations, and focus first on the *Z* personality trait variable in Fig 1, and the *X* strategy variables next. As indicated above, we do so because these are central to the modern managerial theory of firm growth (with *V* variables only included as controls). We note that Egocentrism is ranked as the seventh most important feature. Moreover, the Contribution of Egocentrism is.02 (see Table 3). Although the individual conditional expectation line in the one-way partial dependence plot does not provide a clear direction of the relationship (see Online Technical Appendix 5 in S1 Appendix), we follow the evidence provided by the other outputs. This gives our straightforward *main effect* (D.8) prediction regarding a potentially important characteristic of managers:

*H1*. *Egocentrism is positively associated with firm growth*.

Next, we turn to possible causal-theoretical interpretations of the ‘interactions’ identified in the MFI output. By way of illustration, we develop four alternative models, to be comparatively tested in Step 4 using traditional regression techniques, arguing in favor of (i) full mediation, (ii) partial mediation, (iii) moderation, or (iv) mediated moderation. Here, we adopt a behavioral strategy lens, focusing on possible relationships between personality (*Z*) and strategy (*X*) variables, and how these may affect firm growth performance in conjunction. Table 3 lists the RFA-identified ‘interactions’ per focal variable in descending frequency. We observe three potentially interesting behavioral strategy ‘interaction’ patterns:

1. Pattern 1—Egocentrism most frequently ‘interacts’ with Customer Intimacy and Operational Excellence, but not with Product Leadership.
2. Pattern 2—Operational Excellence and Product Leadership most frequently ‘interact’ with other strategies only, but not with Egocentrism.
3. Pattern 3—Operational Excellence most frequently ‘interacts’ with Egocentrism, but not with any other strategy.

The retroduction step involves theorizing about what this set of three pattern findings may suggest in terms of potential association and causality.

Our first theoretical interpretation revolves around *mediation*. In behavioral strategy, the assumption is that *Z* attributes of key decision-makers affect strategy-related *X* variables. This assumption in behavioral strategy contradicts the assumption we make in our causal model (see Fig 1), in which X cannot be a descendant of Z. Our co-duction methodology distinguishes among what the behavioral strategy argues in theory, what our causal model assumes in practice, and what the data suggest in reality.

Combining patterns 1 and 3, we observe that Egocentrism is only consistently MFI-associated with Operational Excellence, and not with Customer Intimacy or Product Leadership. We note that we could also take RFA output to explicitly identify candidates for a prediction regarding the absence of potentially influential ‘interaction’ effects of a key personality characteristic of the entrepreneur (an X-variable in Fig 1) with well-known strategies (Z-variables in Fig 1). This is in line with the plea to be more explicit about null findings in management and organization research (see, e.g., [72]), as knowledge regarding null findings (and opposite findings) are important to promote scientific progress and to reduce research waste [73]. However, to save space, we refrain from doing so.

Through this mediation lens, the argument then is that Egocentrism is positively associated with Operational Excellence, with the latter having an impact of firm growth. This assumes that the effect of personality on performance runs through personality’s impact on a specific strategy. The first part of the mediation path is what Boone, De Brabander, and van Witteloostuijn [74] refer to as the strategy preference effect, and the second the strategy performance effect. In causality terms, this implies the prediction that a key decision-maker with a specific personality trait is more likely to adopt a specific strategy, with the strategy subsequently impacting performance. Hence, we suggest the following full *mediation effect* (D.9):

*H2 (full mediation)*. *The effect of Egocentrism on firm growth fully runs through the positive association of Egocentrism with Operational Excellence*.

Fig 4a visualizes H2 for the case of full mediation. Below, we will specify a separate hypothesis for partial mediation.

Our second theoretical interpretation involves *moderation*. Through a moderation lens, this suggests that the positive effect of Egocentrism on firm growth, as predicted in Hypothesis 1, is particularly strong if the SME implements an Operational Excellence strategy. This relates to what Boone et al. [74] have coined the strategy implementation effect. That is, a key decision-maker with a specific personality trait (here, Egocentrism) is particularly good at implementing a specific strategy (here, Operational Excellence), because the behavioral and motivational characteristics associated with that specific trait fit particularly well with what is needed to implement that specific strategy well. Hence, we expect the following *moderation effect* (D.9):

*H3 (moderation)*. *The effect of Egocentrism on firm growth is positively moderated by Operational Excellence*.

The net effect on firm growth is ambiguous, as the Contribution metric suggests that the main effect of Egocentrism is positive (Contribution = .02; cf. Hypothesis 1), but that of Operational Excellence is negative (Contribution = -.04). H3 is visualized in Fig 4b.

On top of the predictions implied by H1 to H3, two further possibilities are worth considering. First, if we combine a mediated and a direct effect of Egocentrism on firm growth, we would have partial mediation. In that case, Egocentrism’s main effect (H1) would work together with its mediation impact running through Operational Excellence (H2), implying that both causal chains operate side by side. Second, similarly, both mediation (H2) and moderation (H3) could operate jointly. This implies mediated moderation, where Egocentrism comes with strategy preference and implementation effect. This gives our final pair of hypotheses, which we refer to as H4 (partial mediation) and H5 (mediated moderation), respectively.

*H4 (partial mediation)*. *The effect of Egocentrism on firm growth partially runs through the positive association of Egocentrism with Operational Excellence*.

*H5*: *(mediated moderation)*. *The effect of Egocentrism on firm growth (a) runs through the positive association of Egocentrism with Operational Excellence and (b) is positively moderated by Operational Excellence*.

For the illustrative purposes of how our co-duction cycle plays out in the context of a concrete vignette, the above set of five hypotheses should suffice. However, note that a close inspection of the ML outcomes could suggest further hypotheses, should we aim here to fully explore the insights from our approach in the context of a full-blown firm growth research paper. For instance, Product Leadership is ranked in the eighth position in terms of Feature Importance (see Fig 3) and the Contribution of Product Leadership is.02 (see Table 3). Moreover, the individual conditional expectation line in the one-way partial dependence plot also points in the direction of a positive relation between Product Leadership and firm growth (see Online Technical Appendix 5 in S1 Appendix). As such, we could add the hypothesis that Product Leadership is positively associated with firm growth. But in the context of what we seek to illustrate here, with the above set of five hypotheses in place, we are ready to take the next step in the co-duction cycle: deduction in the form of hypotheses testing using traditional regression techniques. Combining all hypotheses gives the visualization in Fig 4c, in which all alternatives and hypotheses are nested in an overall model. Note that, for the sake of parsimony and readability, we only include our central control, dependent and independent variables.

### Step 4: Hypotheses testing—Deduction

We *specify* (D.10) using standard parametric Structural Equation Modeling (SEM) as the *technique* (D.11) to examine Step 3’s hypotheses in the deductive Step 4, as we have to estimate sets of potentially correlated equations. We use SEM, as this provides the opportunity to estimate a series of paths that together nicely reflect our set of alternative hypotheses. Of course, generally speaking, what we should do in this deduction step is to adopt a traditional probabilistic statistics strategy that best fits the data, hypotheses, and the overall aim to come as close as we can to identify causalities (cf. [22] Imbens, 2022). But given the low-t dimensionality of our data without any ‘natural treatment’, alternatives (such as two-stage panel econometrics with instruments) are not an option anyway. Note that the ML community is developing algorithms designed to identify causalities, too (e.g., [19, 75]).

As explained above, we decided to work with three splits of our data–a learning and validation subsample for our ML process pipeline in the induction Step 2, and a third updated and separate sample to examine our hypotheses with traditional regression on data unseen by the ML algorithm in our deduction Step 4. In so doing, we adopt the research process pipeline standard in the ML tradition to avoid any data leakage that would inflate later-stage fit statistics—and thus, in our case inflated SEM estimates. Specifically, our SEM sample is an update of the ML subsamples’ observations, involving a time window that is moved one year up, ending in 2018 (for SEM) rather than 2017 (for ML). The SEM sample thus contains data that has not been used to train the ML algorithm.

Barrett [76] suggests that the Chi-squared statistic is the “only substantive test of fit for SEM”, but also points out that this metric is very sensitive to sample size. For models with about 75 to 200 cases, the Chi-squared is generally a reasonable statistic of fit. However, when more than 400 cases are used, the Chi-squared statistic is almost always statistically significant [77]. This is why the Chi-squared statistic has been all but abandoned for estimating the goodness-of-fit of SEMs [78], and is certainly not applicable to our dataset with an *n* of 8,163. Instead, Kenny [77] suggests using the RSMEA and TLI as alternatives. In the following, we will report both, and also the CFI and *R*^2^ as additional statistics. There is considerable debate on the thresholds of these metrics that would indicate an acceptable fit [79]. For the purpose of this paper, we will stick with the basic recommendations. Browne and Cudeck [80] suggest that the maximum RSMEA value is 0.10, while the minimum value for the TLI and CFI is 0.9 [81, 82].

Below, we step-by-step discuss the SEM estimates related to our alternative hypotheses. Given that the scale of our independent variables varies, we follow Grace et al. [83] and report standardized estimates. This permits evaluation of the relative importance of variables in the models. In Table 4, we provide the key estimates that directly relate to our four alternative models, as represented by H2-5, plus the main effect benchmark H1. For the sake of brevity, we refer to the Online Appendix 9 in S1 Appendix for the full SEM results, which include a set of six control variables and all associated paths.

**Table 4:** SEM regression for H1-5.

SEM paths	H1	H2 & H4	H3	H5
Egocentrism → Firm Growth	See the estimates in italics for H2.	10.879	7.552	8.503
		(5.515)	(5.980)	(5.960)
		[.049]	[.207]	[.154]
Egocentrism → Operational Excellence		.088		.090
		(.0006)		(.0006)
		[.000]		[.000]
Operational Excellence → Firm Growth		2.141	-19.230	2.044
		(11.749)	(18.76)	(11.575)
		[.855]	[.306]	[.862]
Egocentrism*Operational Excellence → Firm Growth			198.218	89.883
			(135.771)	(84.947)
			[.144]	[.290]
TLI		.835	.992	.856
CFI		.871	.993	.891
RMSEA		.104	.070	.105
R^2^		.072	.094	.081

Notes: (1) Total Assets (*t*-1), Working Capital (*t*-1), Current Ratio (*t*-1), Solvency Ratio (*t*-1) and Number of Employees (*t*-1) are included as control variables, but Total Equity (t-1) was excluded given its high and significant correlation with Total Assets (t-1); (2) The columns give the *ß* estimates for the models associated with the test of the hypothesis/hypotheses in the column heading, with standard errors in parentheses and *p*-values in squared brackets; and (3) *n* = 8,163.

Note that the output offers support for—and *verification* (*D*.*12*) of—H1: Egocentrism is positively associated with Firm Growth, if estimated as a main effect in the mediation model (*p* = .049). The effect of Egocentrism is pronounced. An increase from completely not Egocentric (0) to completely Egocentric (1) is associated with a 10.9%-point increase in Firm Growth. It should be noted, however, that the mean value of Egocentrism is.15 with a standard deviation of.04. The maximum observed value for Egocentrism in the sample is only.6. The standardized effect sizes, then, give a better view of the relative importance of the variables in the model. Although Egocentrism remains an important predictor, in terms of effect size this trait ranks below Current Ratio (*t*-1) and Number of Employees (*t*-1).

Regarding full or partial mediation (H2 and H4), the model just fails the minimum thresholds for the TLI and CFI, but results in an acceptable value for the RSMEA. Hence, the model gives a moderate fit with the data, at best. The *p* values for the estimates for the effects of Egocentrism and Operational Excellence on Firm Growth are *p* = .049 and *p* = .855, respectively, with the (standardized) effect estimates being larger than.01. The 95% confidence interval for the estimate of the effect of Egocentrism on Operational Excellence does not cross zero. This would suggest that Egocentrism does affect Firm Growth and Operational Excellence, but that this effect is not mediated by Operational Excellence. In all, for now, we have to decide against both full mediation and partial mediation (H2 and H4).

Turning to moderation (H3), this model achieves acceptable scores across all three goodness-of-fit statistics. The *p* value for the Egocentrism*Operational Excellence coefficient is too high, at *p* = .144. Additionally, the Egocentrism–Firm Growth and Operational Excellence–Firm Growth paths are both insignificant at *p* = .207 and *p* = .306, respectively. Note that the mean value for Operational Excellence is.015 and the standard deviation is.02. This explains the large effect size (198% point increase in Firm Growth) of the interaction term, which multiplies Egocentrism and Operational Excellence, and thus results in an even lower mean. The marginal effects plot (see Online Appendix 10 in S1 Appendix) shows that the Egocentrism*Operational Excellence moderation effect is significant for values of Operational Excellence above.015. This, in combination with the considerable effect sizes and the good model fit, provides support for H3.

Finally, with regards mediated moderation (H5), this model gives a mediocre fit with the data, as all three test statistics score just below their minimum threshold. Although the Egocentrism—Operational Excellence path is significant (*p* = .000), all other paths are not, mostly achieving moderate (standardized) effect sizes. For instance, the Operational Excellence–Firm Growth path is not significant and has a very small effect estimate. Inspecting the marginal effect plot reveals that the Egocentrism*Operational Excellence moderation effect is significant for values above.01. In all, the mediation part of the model does not fit with the data, as in the mediation model above, but the moderation part does, albeit only for a limited number of observations, as in the moderation model above. Hence, for now, we have to decide against the moderated mediation hypothesis (H5).

In all, reflecting upon our set of findings, as inspired by Step 2’s inductive pattern-identification inductive ML analysis, and the conceptually developed hypotheses in the retroduction Step 3, suggests that the ‘interaction’ examined in Step 2 comes closer to moderation of personality (i.e., Egocentrism) and strategy (i.e., Operational Excellence), and not (full or partial) mediation, for the limited number of SMEs scoring high on Operational Excellence, in combination with a forceful Egocentrism personality main effect for a much larger number of SMEs as the ‘best’ explanation of firm growth. Note that above we tried to avoid the use of strict testing language, as model comparison alone cannot provide evidence that distinguished mediation from moderation (but see [84]). Below, in the final abductive Step 5 of our co-duction cycle, we reflect on what this set of findings might imply for theory.

### Step 5: Improved theorization—Abduction

The final step in the co-duction cycle is to carefully interpret the set of findings in Step 4 to theorize the underlying *causal mechanisms* (D.13), abductively searching for the best explanation of our set of results. We do so for the main (in)significant results regarding our set of hypotheses, as produced by our deductive multivariate regression analyses in Step 4. The first finding involves the positive main effect of an entrepreneur’s egocentrism on firm growth (H1): The higher an entrepreneur’s egocentrism, the higher the likelihood of above-average firm growth, *ceteris paribus*. Here, the underlying argument may be that egocentrism is positively associated with hubris [85], and hence is positively correlated with an empire-building ambition [86]. After all, we know from the literature that egocentrism comes with a feeling of discretion, risk-taking behavior, and power motivation [87], which all feed into an intrinsic motivation to grow large [88]. In the context of an SME, such an ambition can be realized by aiming for high firm growth.

The second finding involves the null result regarding the mediation of an entrepreneur’s egocentrism impacting firm growth via strategy. The strategy that stands out in the ML analysis is operational excellence. However, the positive effect of egocentrism on firm growth does not run through operational excellence, not even partially. This is not because egocentrism does not come with a preference for operational excellence. It does, as the association of egocentrism with operational excellence is positive. However, this strategy is not related to firm growth. We argue that this finding in tandem with egocentrism’s forceful main effect implies a strong version of the behavioral theory of strategy, with the personality effect crowding out the impact of strategy *per se*. A reason for this might be that an egocentric personality comes with (very) high self-confidence, if not over-optimism, which buffers the individual’s behavior against external mishaps or internal push-backs [89]. And, in line with the above, an egocentric entrepreneur may engage in risky investment in firm growth anyway so as to build an empire and accumulate power, not accepting that whatever strategy, operational excellence or otherwise, is a hurdle along the path to greatness, featuring high internal locus of control [90], and being highly opinionated and persevering [91].

The third finding relates to the significant and positive moderation effect of an entrepreneur’s egocentrism with operational excellence, but only for the case of extremely high scores for this strategy. That is, having a clear-cut competitive strategy profile of the operational excellence kind further stimulates the growth-stimulating effect of the egocentric personality of the SME’s leader. Perhaps, this is due to the opportunistic side of an egocentric personality [92]. Having a clear-cut operational excellence strategy implies that processes and structures are in place that define practices and routines associated with such a strategy, binding the employees and the entrepreneur to specific ‘ways of doing things’, producing a certain degree of organizational rigidity [93]. With such a clear-cut operational excellence strategy, the egocentric entrepreneur’s hands are tied to the associated practices and routines, like Odysseus to the mast of his ship, leaving ample room to engage in complementary empire-building activities–for instance, in the form of galvanizing leadership and extensive external stakeholder management.

A key limitation of our study is that we only have data for larger SMEs from the Dutch-speaking part of Belgium. So, regarding external validity, further work is needed to explore the *boundary conditions* of our findings (*D*.*14*), collecting data in other countries, as well as for both smaller and larger enterprises. Moreover, the limited set of explanatory variables in our dataset suggests an extensive future research *agenda* (*D*.*15*), as does the need to further explore the suggested causal mechanisms. This underlines the remark made above that one co-duction cycle is rarely enough to reach the end of the road toward better theories. In practice, we need a chain of cycles to achieve this aim, step-by-step reducing unexplained variance whilst identifying further causal mechanisms. Regarding the latter, the next cycle might well involve other designs and methods than the ones included in the five steps of our co-duction cycle, as causal inference requires designs and associated methods that come closer to the Holy Grail of a randomized controlled trial, such as data that involve a field or natural experiment (cf. [94]). We return to this issue below.

## Conclusion

We introduce a methodological research process cycle of what we refer to as one involving “*co-duction*”, combining five steps—*reduction*, *induction*, *retroduction*, *deduction*, and *abduction*. Our co-duction cycle is appropriate in cases where high unexplained variance can be reduced by analyzing high-dimensional datasets large enough to meaningfully apply ML techniques. We note that the dataset’s dimensions do not have to be very large, actually, as the argument that ML only works with Big Data is an urban myth (see [9]).

The co-duction cycle builds on the methodologies and methods that are advocated by pioneers of artificial intelligence. We demonstrate the use of the cycle’s five steps and the associated methods with a vignette with real-world observational data in which we extend earlier empirical work [21] that applies ML algorithms and statistical analyses to explain or predict firm growth. Importantly, with our vignette (and Online Technical Appendices in S1 Appendix), we hope to clearly illustrate the mechanics of our proposed methodology, including novel metrics and visualizations, so as to provide a menu of guidelines and techniques that can be readily used in future research. We would like to conclude our paper by reflecting upon a few important issues, including limitations suggesting promising future research issues.

The first issue involves the question as to what a cycle of co-duction methods of reasoning, methods, and techniques contributes to the extant literature that already argues in favor of the value added of modern ML techniques (cf. [7]). Recently, for instance, algorithm-assisted theorizing has been advocated as a new approach for theory building in management and organization science research in the form of ML-generated pattern detection that facilitates inductive theorizing [4] and exploratory pattern discovery that assists data-driven inductive theory building [5], relating to ML’s key strength of quantitative pattern detection. However, such algorithm-assisted theorizing would fail to explain much of the variance in the outcome if the prediction model merely reflects a collection of ‘interacting’ determinants without a clear identification of the essential underlying causal mechanisms. We argue that ML-based induction could be combined with regression-based deduction in the context of a co-duction cycle that abductively identifies plausible underlying causal mechanisms. Note that this should not be interpreted as an exclusive association of induction with ML and deduction with traditional statistics–certainly not. After all, ML can also be used deductively, and traditional statistics can be applied to inductively explore data. This limitation of the version of the co-duction cycle developed in the current paper can be inspire future work.

This immediately raises a second important issue: What should follow next on our journey to develop better theories? As we submitted earlier, better theories in the social sciences (a) reduce unexplained variance (of phenomena not associated with dominant randomness) whilst (b) identifying plausible underlying causal mechanisms [with (b) dominating (a) would both objectives be at odds with each other]. Our firm growth vignette illustrates how analyzing high-dimensional observational data by carefully taking sequentially all five steps of our co-duction cycle, applying modern inductive ML pattern-detection techniques first and classic deductive regression methods next, can be instrumental in drastically reducing unexplained variance. However, after one iteration there should be another iteration of a similar or different cycle to further explore the identified patterns and mechanisms. A similar cycle with similar data can be used to examine the boundary conditions of the findings in other countries and enterprises from different size classes. A different cycle, which can take different shapes from what we presented in this paper, can extend the analysis by collecting richer data, adding a series of extra variables that can plausibly be expected to be part of the ensemble of (weak or strong) determinants.

A final reflection is worth making to close the chain of this paper’s reasoning. The methods associated with the co-duction cycle are complementary to the other methods in our research toolkit. As an example, in the vignette, Step 5 has produced specific hypotheses regarding plausible causal mechanisms. Hence, in the next deductive cycle, another design is required that offers the opportunity to deeply probe into causal mechanisms. For instance, data collection with a survey instrument may have to be combined with a panel-type longitudinal follow-up design. Per the recommendation of one of our reviewers, a potential solution to address the issue would involve two approaches. Firstly, delineating a comprehensive causal diagram followed by conducting causal inference, as detailed in the works of Pearl. Secondly, employing the experimental method to empirically test the hypothesized effects using new data sets could be considered.

Another example could be that further inductive case studies are performed to feed into Step 1 of a subsequent co-duction cycle in order to further enrich the selection of potentially important explanatory variables (which then, of course, have to be measured in order to be appended to the current dataset). Note that the iteration of cycles may or may not continue infinitely. On the one hand, the cycles may conclude when the research community agrees that there is a plateau reached in the reduction of unexplained variance, provided that the ‘true’ data generation process is not dominated by randomness. On the other hand, in principle, any theory is a simplification, implying that no theory can be a perfect representation of reality. Through this lens, the sequence of cycles may well continue *ad infinitum*.

We further note that, although it is tempting to set a threshold for a stopping rule at a particular value of explained variance (e.g. 60 or 80 percent), such a threshold will not be universally applicable due the inherent variability in the reducible error across different problems. As kindly pointed out by one of our reviewers, irreducible error is an intrinsic, though often unknown, characteristic of the system under investigation. Moreover, we highlight that full explanation of all variance is not the ultimate aim–enough is enough (which will always be somewhat arbitrary), and an R^2^ of (close to) 100 is likely to manifest overfitting.

To conclude, Sutton and Staw [95] (p. 372) declared that “Lack of consensus on exactly what theory is may explain why it is so difficult to develop strong theory in the behavioral sciences. Reviewers, editors, and other audiences may hold inconsistent beliefs about what constitutes theory and what constitutes strong versus weak theory.” Yet, Suddaby [96], building on Bacharach [97], contested that, “There is, perhaps surprisingly, considerable consensus on what theory is: theory is simply a way of imposing conceptual order on the empirical complexity of the phenomenal world.” We add one possible route to resolve the tension between these two contrasting views on what theory is (not), by advancing a methodology for building better theories that we refer to as the co-duction cycle. In this cycle, we combine the well-known strengths of classic deductive regression methods with those of modern inductive ML techniques to analyze high-dimensional observational data. Together, combining the insight from both sets of analyses leads abductively to the identification of plausible underlying causal mechanisms that may lead to more better theories. Hence, we believe that by applying our co-duction cycle iteratively and systematically, the social sciences can produce theories that deepen our understanding of complex phenomena.

## Supporting information

S1 Appendix Online technical appendices. (PDF)

S1 Data Contact information. (PDF)
