# Supplementary material for: Data science and automation in the process of theorizing: Machine learning’s power of induction in the co-duction cycle
Source: PLoS One. 2024 Nov 4;19(11):e0309318. doi: 10.1371/journal.pone.0309318 (PMC11534228; doi:10.1371/journal.pone.0309318)
Supplement: S1 Appendix — (PDF) [file pone.0309318.s001.pdf]

ONLINE TECHNICAL APPENDICES

1. Correlation matrix

	Growth	Total Assets (*t*)	Working Capital (*t*)	Total Equity (*t*)	Current Ratio (*t*)	Solvency Ratio (*t*)	# of Employees (*t*)	Total Assets (*t*-1)	Working Capital (*t*-1)	Total Equity (*t*-1)	Current Ratio (*t*-1)	Solvency Ratio (*t*-1)	# of Employees (*t*-1)	Egocentrism	Customer Intimacy	Pro[illegible] Lead[illegible]
**Growth**																
**Total Assets (*t*)**	-0.01															
**Working Capital (*t*)**	-0.00	-0.14														
**Total Equity (*t*)**	-0.01	0.74***	0.28													
**Current Ratio (*t*)**	-0.02	-0.00	0.02	0.01												
**Solvency Ratio (*t*)**	0.02	0.00	0.01	0.01	0.18											
**Number of Employees (*t*)**	0.01	0.41**	-0.24**	0.25	-0.01	-0.00										
**Total Assets (*t*-1)**	-0.01	0.94***	-0.10	0.85***	-0.00	0.00	0.4**									
**Working Capital (*t*-1)**	-0.01	0.11	0.94***	0.45*	0.02	0.01	-0.1	0.15								
**Total Equity (*t*-1)**	-0.01	0.80***	0.38	0.98***	0.01	0.01	0.22	0.84***	0.56**							
**Current Ratio (*t*-1)**	-0.01	-0.00	0.02	0.01	0.51*	0.09	-0.01	0.0	0.02	0.01						
**Solvency Ratio (*t*-1)**	0.02	-0.00	0.01	0.0	0.12	0.46***	-0.0	-0.0	0.01	0.00	0.08					
**# of Employees (*t*-1)**	0.02	0.34**	-0.24**	0.21	-0.02	-0.01	0.93***	0.42**	-0.11	0.20	-0.01	-0.01				
**Egocentrism**	-0.0	-0.01	-0.00	-0.01	0.13	0.01	-0.01	-0.01	-0.00	-0.01	0.06	0.01	-0.01			
**Customer Intimacy**	0.01	-0.01	-0.01	-0.01	-0.03	-0.0	0.00	-0.01	-0.00	-0.01	-0.02	-0.01	0.00	-0.05		
**Product Leadership**	0.02	-0.00	-0.00	0.00	0.04	0.01	0.00	-0.00	-0.00	0.00	0.02	0.00	0.00	0.08	0.02	
**Operational Excellence**	0.00	-0.00	0.00	0.00	0.02	0.0	0.00	-0.00	-0.00	0.00	0.01	0.00	0.00	0.02	-0.01	-0[illegible]

* p < 0.1   ** p < 0.05   *** p < 0.01

2. LDA Tuning

Hyper-parameter search space	Optimized hyper-parameter setting
Components (1 – 1000)	Components (100)
Learning Decay (0.001 – 1)	Learning Decay (0.7)
Alpha (0.01 – 0.3)	Alpha (0.2)
Eta (0.01 – 0.3)	Eta (0.5)

We used Random Search to find the optimal values for the LDA model. We evaluated on perplexity, which is a measurement of how well a probability model predicts a sample [1]. We tuned four hyper-parameters: (1) Components; (2) Learning Decay; (3) Alpha; and (4) Eta. Components refer to the number of topics, and Learning Decay to the decrease in the learning rate over training iterations. Alpha determines the prior distribution of topic weights over each document, and Eta the prior distribution of word weights over each topic. For each of the hyper-parameters, we specified the search space in a linear interval of 50 steps between the minimum and maximum values outlined above. The number of randomly chosen combinations of hyper-parameters was set to 100. With this setting, Random Search finds a solution within 5% of the global optimal solution 99% of the time [2]. We favored Random Search over Grid Search because the former finds "better models in most cases and require[s] less computational time" (ibid., p. 302). We selected the optimized hyper-parameters following the 1-SE rule to "choose the simplest model whose accuracy is comparable with the best model" [3] (p. 11). More concretely, we rounded the hyper parameters to the nearest integer (for Components) or first decimal (for the other hyper-parameters). The optimized model achieved a perplexity of 1899.65.

3. Random Forest Hyper-parameter turning

Hyper-parameter search space	Optimized hyper-parameter setting
Bootstrap (True, False)	Bootstrap (True)
Maximum Depth (1 – 100)	Maximum Depth (90)
Number of Trees (1 – 500)	Number of Trees (100)
Minimum Samples per Leaf (1 – 50)	Minimum Samples per Leaf (2)
Minimum Samples for Split (2 – 50)	Minimum Samples for Split (2)
Maximum Number of Features (Auto, Sqrt, Log2, None)	Maximum Number of Features (Auto)

We used a similar Random Search to find the optimal values for the Random Forest Analysis (RFA). We used the mean squared error as the loss function, and evaluated on R^2. We tuned six hyper-parameters: (1) Bootstrap; (2) Maximum Depth; (3) Number of Trees; (4) Minimum Samples per Leaf; (5) Minimum Samples for Split; (6) and Maximum Number of Features. Bootstrap determines if the data for each tree in the Random Forest is drawn with – or without – replacement. Maximum Depth involves the maximum number of each tree in the Random Forest. Number of Trees is the number of trees in the Random Forest. Minimum Samples for Split implies the minimum number of samples required to split a node in a tree. Minimum Samples per Leaf determines the minimum number of samples in a node after a split. Maximum Number of Features is the maximum number of independent variables per tree in the Random Forest. The optimized hyper-parameters were selected using the strategy outlined under "LDA Tuning". The optimal settings were then applied to an RFA, which was fitted on the training and test dataset combined. The optimized model achieved an R^2 of 0.63.

4. Feature Importance

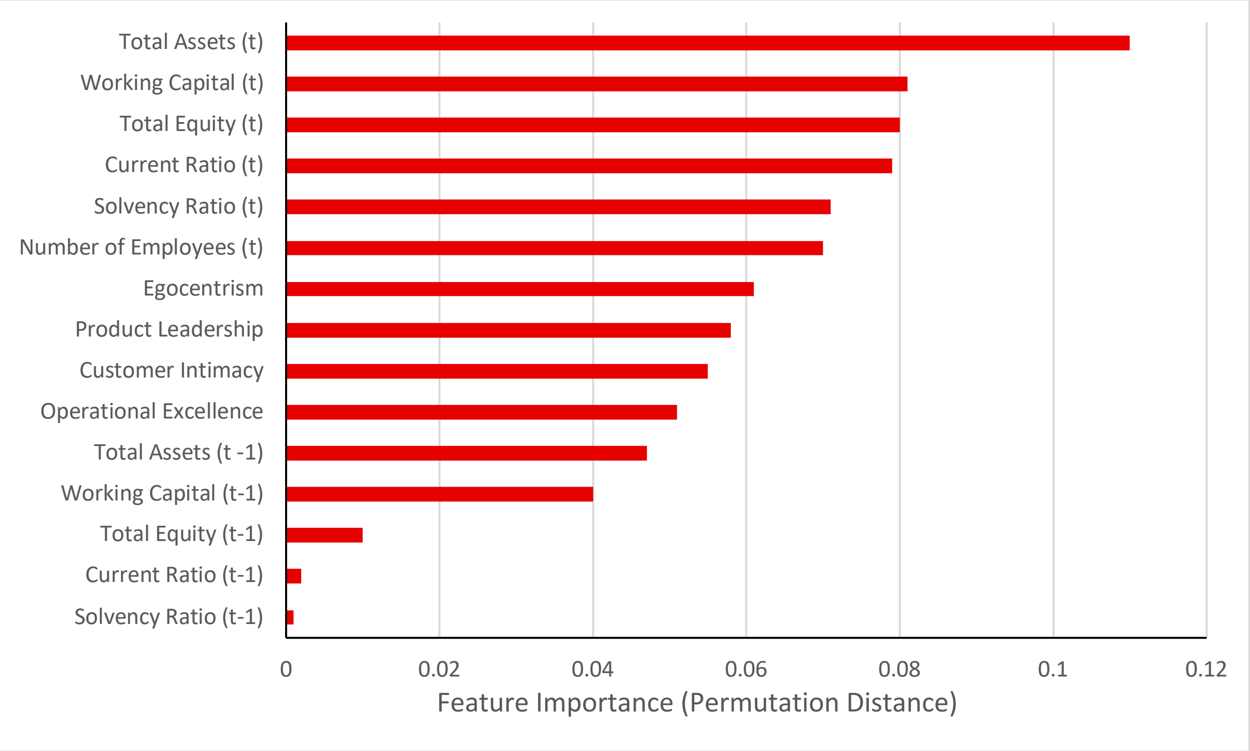

The Feature Importance graph shows the relative contribution of each independent variable (or feature, in RF terminology) on the Random Forest Analysis's predictions. Feature Importance says nothing about the direction of that contribution, but does provide insight in the relative importance of the independent variable involved. We computed the Feature Importances using permutation distance. Permutation Feature Importance measures the increase in the prediction error of the model after manipulating the feature values [4]. As such, Feature Importance measures take account of all interactions between independent variables in the model. This means that the permutation Feature Importance captures both the main feature effect and the interaction effects on model performance. This is also a disadvantage because the importance of the interaction between two features is included in the Importance measurements of both features [5].

5. One-way partial dependence plots with individual conditional expectations

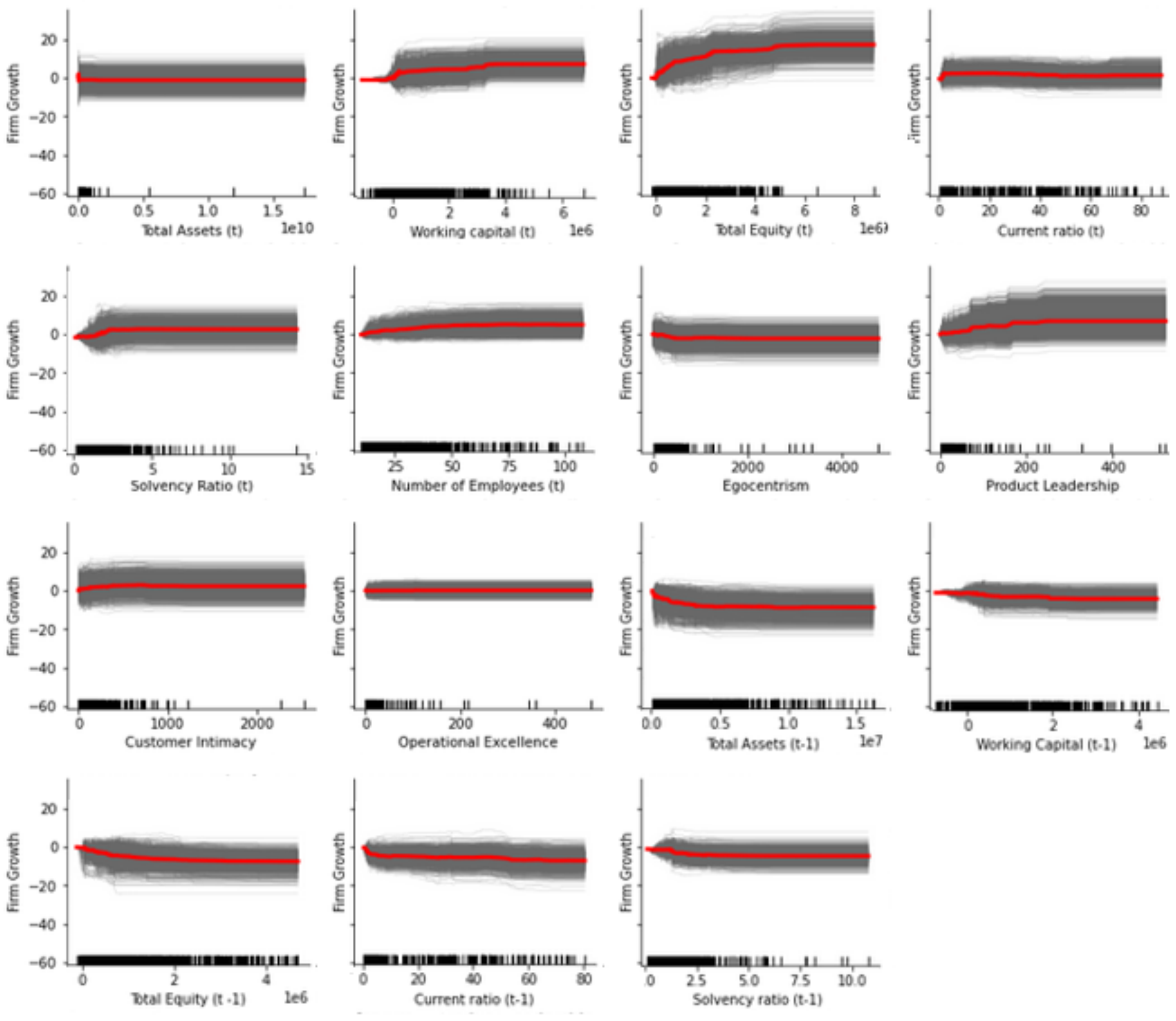

The graph above shows the partial dependence plots with individual conditional expectations for the 15 most important independent variables in the Random Forest. A one-way partial dependence plot (PDP) shows the marginal effect of one feature on the predicted outcome of a Random Forest [6]. A partial dependence plot helps to identify the type of relationship between the independent and the dependent variable. Individual conditional expectation (ICE) plots display one line per instance that visualizes how the instance's prediction changes when a feature changes [7]. The PDP (in red) is the average of the ICE lines (in grey). We used a sub-sample of 900 records to compute the graphs. Where the Feature Importance metric provides insight into the relative importance of the independent variable involved, the PDP indicates the relation between the independent and the dependent variable. It is important to note that PDP and ICE operate under the assumption of independence: That is, it is assumed that the independent variable for which the PDP is computed does not correlate with any other independent variable. As is evident from the correlation matrix, this assumption is violated in our dataset. Hence, caution is necessary when interpreting the PDP plots.

6. Feature Contributions and top-three feature interactions (MFIs)

#	Variable	MFI	Contribution
1	Total Assets (*t*)	3,11,13	0.09
2	Working Capital (*t*)	3,13,11	-0.02
3	Total Equity (*t*)	1,11,13	0.77
4	Current Ratio (*t*)	3,13,11	-0.07
5	Solvency Ratio (*t*)	3,13,15	-0.02
6	Number of Employees (*t*)	3,1,11	-0.03
7	Egocentrism	3,4,11	0.02
8	Product Leadership	1,4,14	0.02
9	Customer Intimacy	3,1,11	0.03
10	Operational Excellence	3,1,11	-0.04
11	Total Assets (*t* -1)	3,13,1	0.15
12	Working Capital (*t*-1)	3,11,13	-0.02
13	Total Equity (*t*-1)	3,11,1	0.13
14	Current Ratio (*t*-1)	3,5,13	-0.04
15	Solvency Ratio (*t*-1)	3,1,11	0.00

This table lists the mean Contributions and Most Frequent Interactions (MFIs) for each of the 15 most important independent variables in our Random Forest. For a tree in the Random Forest, the Contribution is the difference between the value at the present node and the previous one [8]. It thus provides an indication of the impact of a particular split in the tree on the prediction. The table above shows the mean feature Contribution for the cases with a mean absolute percentage error smaller than 10%, across all trees in the Random Forest for all splits that involve a particular variable. The MFI is an adaptation of the mutual feature interaction introduced by [9]. For the cases with an absolute percentage error smaller than 10%, we compute the number of times two features appear on the same path for all trees in the Random Forest. It is important to note that the feature Contributions are computed across all trees and for all records, whereas the PDP were derived from a sub-sample of records. As such, there may be differences between the two in terms of the direction of the relation between the independent variables and dependent variable.

7. Two-way partial dependence plots

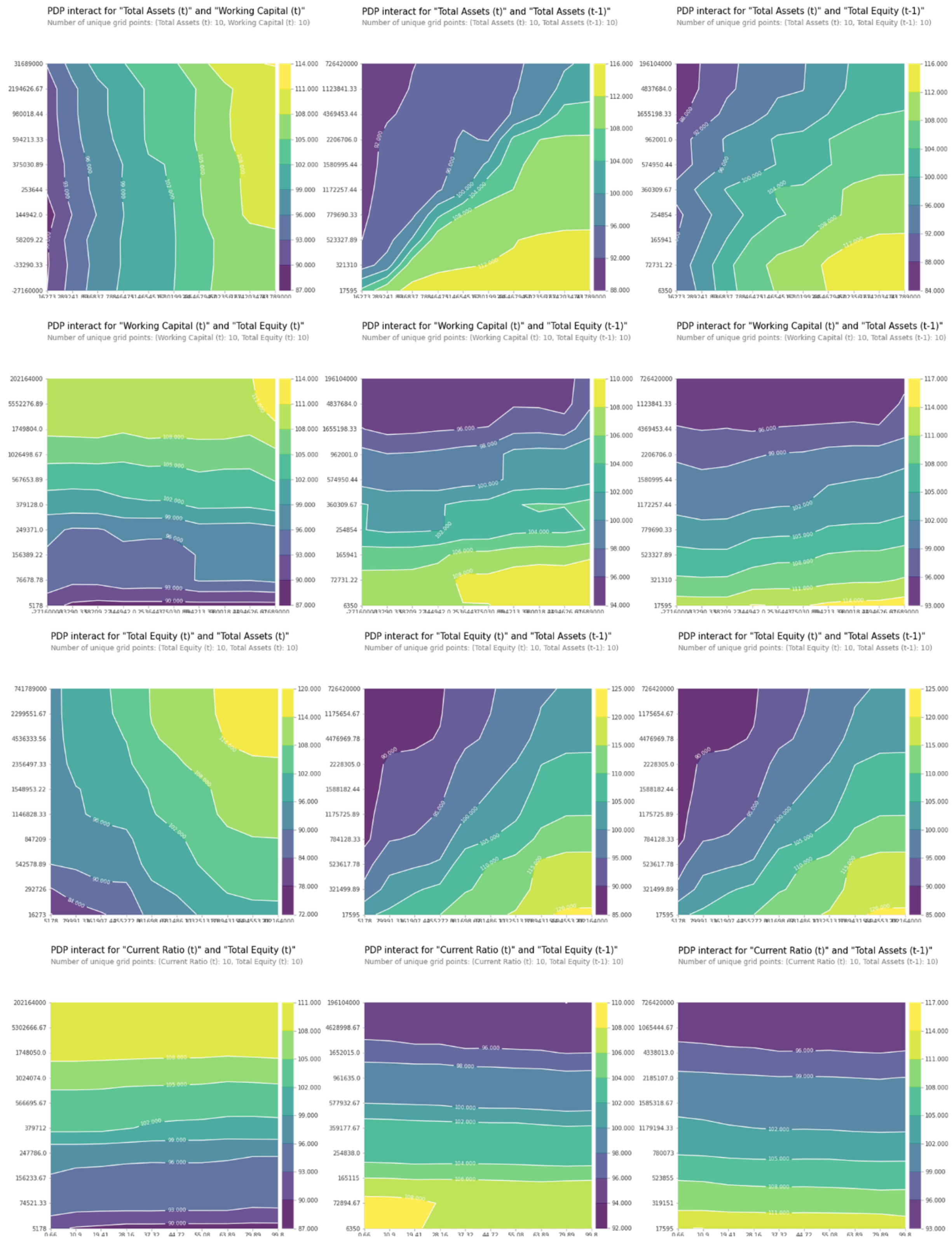

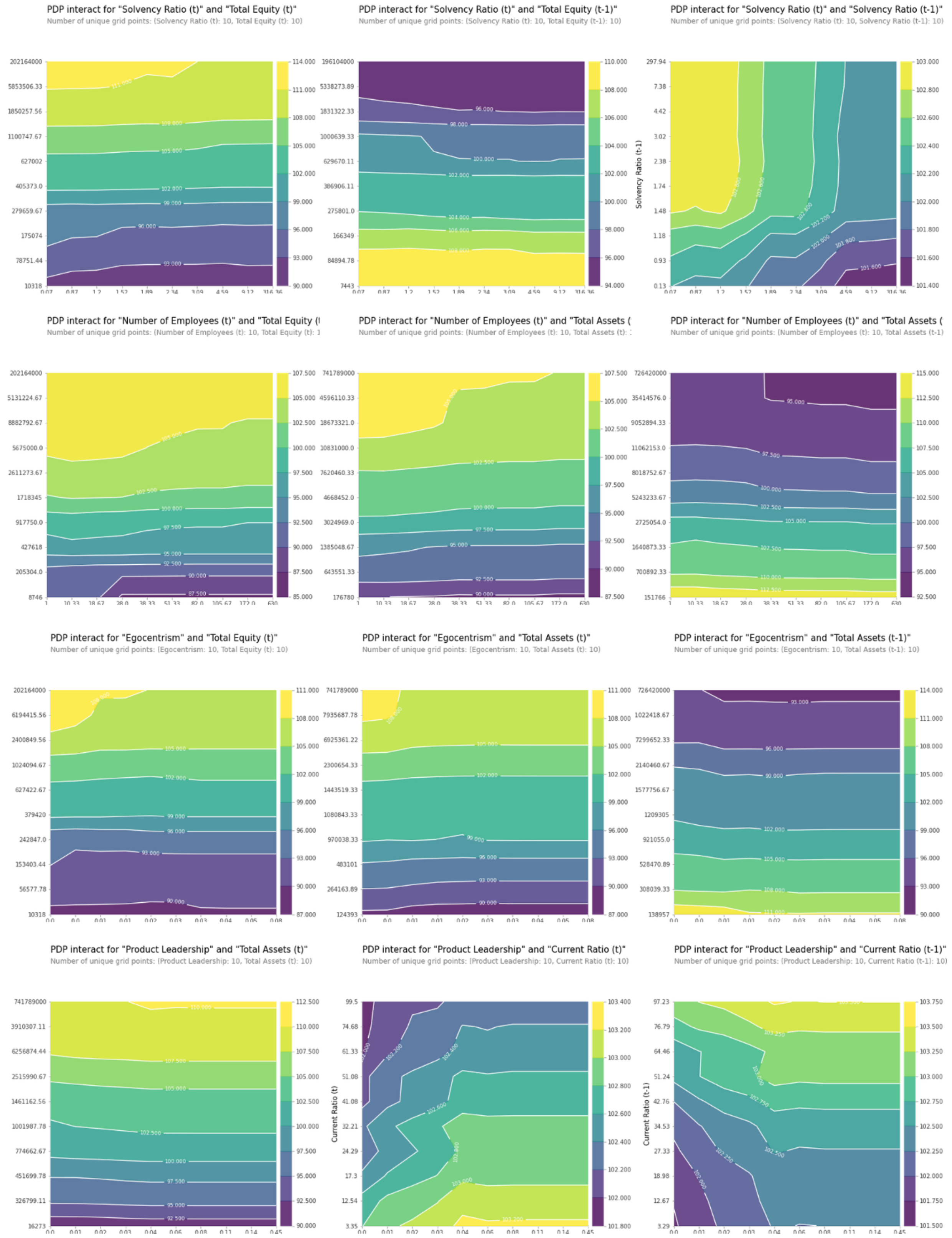
PDP interact for "Solvency Ratio (t)" and "Total Equity (t)"
Number of unique grid points: (Solvency Ratio (t): 10, Total Equity (t): 10)
PDP interact for "Solvency Ratio (t)" and "Total Equity (t-1)"
Number of unique grid points: (Solvency Ratio (t): 10, Total Equity (t-1): 10)
PDP interact for "Solvency Ratio (t)" and "Solvency Ratio (t-1)"
Number of unique grid points: (Solvency Ratio (t): 10, Solvency Ratio (t-1): 10)
Solvency Ratio (t-1)
PDP interact for "Number of Employees (t)" and "Total Equity (
Number of unique grid points: (Number of Employees (t): 10, Total Equity (t): 1
PDP interact for "Number of Employees (t)" and "Total Assets (
Number of unique grid points: (Number of Employees (t): 10, Total Assets (t):
PDP interact for "Number of Employees (t)" and "Total Assets (
Number of unique grid points: (Number of Employees (t): 10, Total Assets (t-1)
PDP interact for "Egocentrism" and "Total Equity (t)"
Number of unique grid points: (Egocentrism: 10, Total Equity (t): 10)
PDP interact for "Egocentrism" and "Total Assets (t)"
Number of unique grid points: (Egocentrism: 10, Total Assets (t): 10)
PDP interact for "Egocentrism" and "Total Assets (t-1)"
Number of unique grid points: (Egocentrism: 10, Total Assets (t-1): 10)
PDP interact for "Product Leadership" and "Total Assets (t)"
Number of unique grid points: (Product Leadership: 10, Total Assets (t): 10)
PDP interact for "Product Leadership" and "Current Ratio (t)"
Number of unique grid points: (Product Leadership: 10, Current Ratio (t): 10)
Current Ratio (t)
PDP interact for "Product Leadership" and "Current Ratio (t-1)"
Number of unique grid points: (Product Leadership: 10, Current Ratio (t-1): 10)
Current Ratio (t-1)

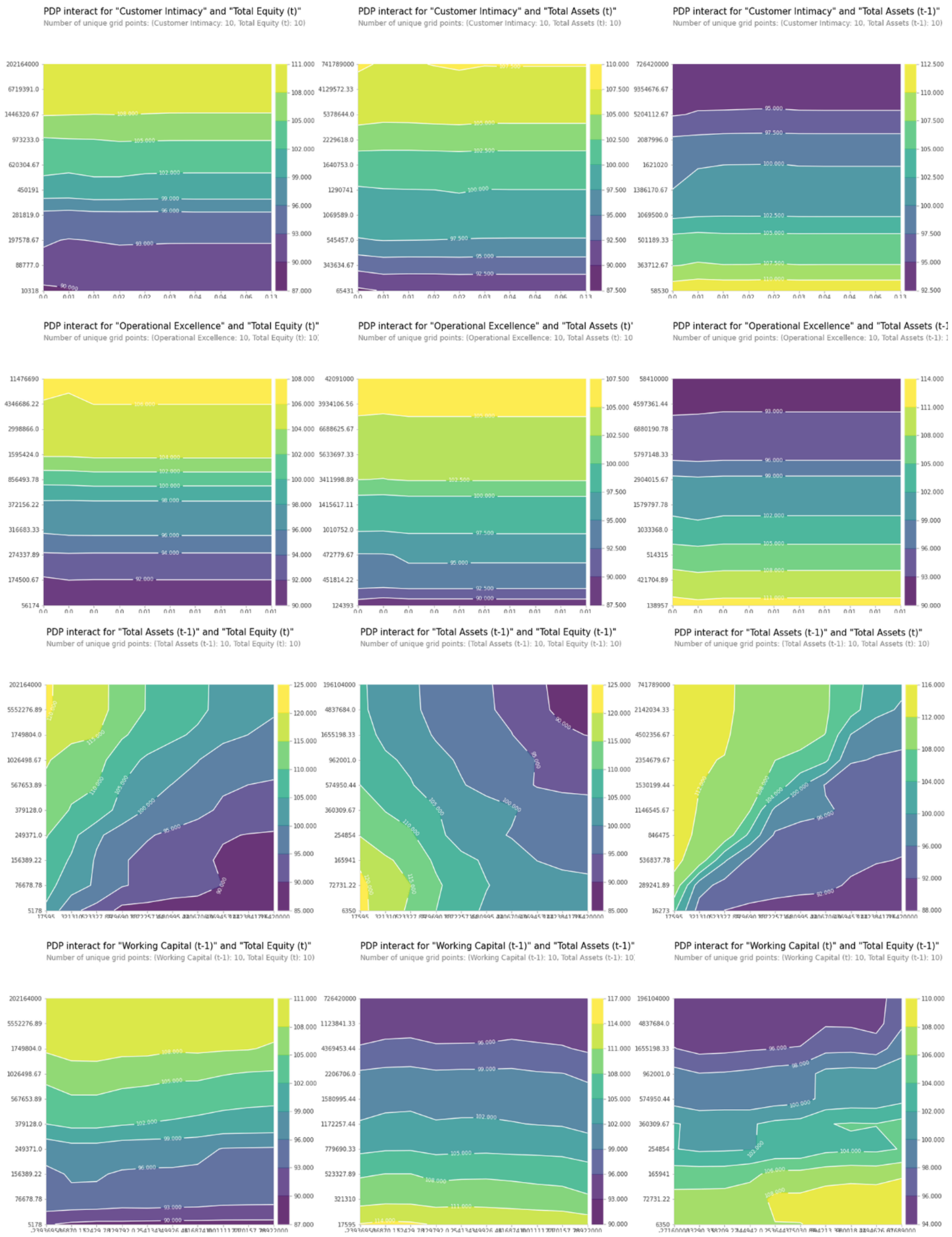
PDP interact for "Customer Intimacy" and "Total Equity (t)"
Number of unique grid points: (Customer Intimacy: 10, Total Equity (t): 10)
PDP interact for "Customer Intimacy" and "Total Assets (t)"
Number of unique grid points: (Customer Intimacy: 10, Total Assets (t): 10)
PDP interact for "Customer Intimacy" and "Total Assets (t-1)"
Number of unique grid points: (Customer Intimacy: 10, Total Assets (t-1): 10)
PDP interact for "Operational Excellence" and "Total Equity (t)"
Number of unique grid points: (Operational Excellence: 10, Total Equity (t): 10)
PDP interact for "Operational Excellence" and "Total Assets (t)'
Number of unique grid points: (Operational Excellence: 10, Total Assets (t): 10
PDP interact for "Operational Excellence" and "Total Assets (t-1
Number of unique grid points: (Operational Excellence: 10, Total Assets (t-1):
PDP interact for "Total Assets (t-1)" and "Total Equity (t)"
Number of unique grid points: (Total Assets (t-1): 10, Total Equity (t): 10)
PDP interact for "Total Assets (t-1)" and "Total Equity (t-1)"
Number of unique grid points: (Total Assets (t-1): 10, Total Equity (t-1): 10)
PDP interact for "Total Assets (t-1)" and "Total Assets (t)"
Number of unique grid points: (Total Assets (t-1): 10, Total Assets (t): 10)
PDP interact for "Working Capital (t-1)" and "Total Equity (t)"
Number of unique grid points: (Working Capital (t-1): 10, Total Equity (t): 10)
PDP interact for "Working Capital (t-1)" and "Total Assets (t-1)"
Number of unique grid points: (Working Capital (t-1): 10, Total Assets (t-1): 10)
PDP interact for "Working Capital (t)" and "Total Equity (t-1)"
Number of unique grid points: (Working Capital (t): 10, Total Equity (t-1): 10)

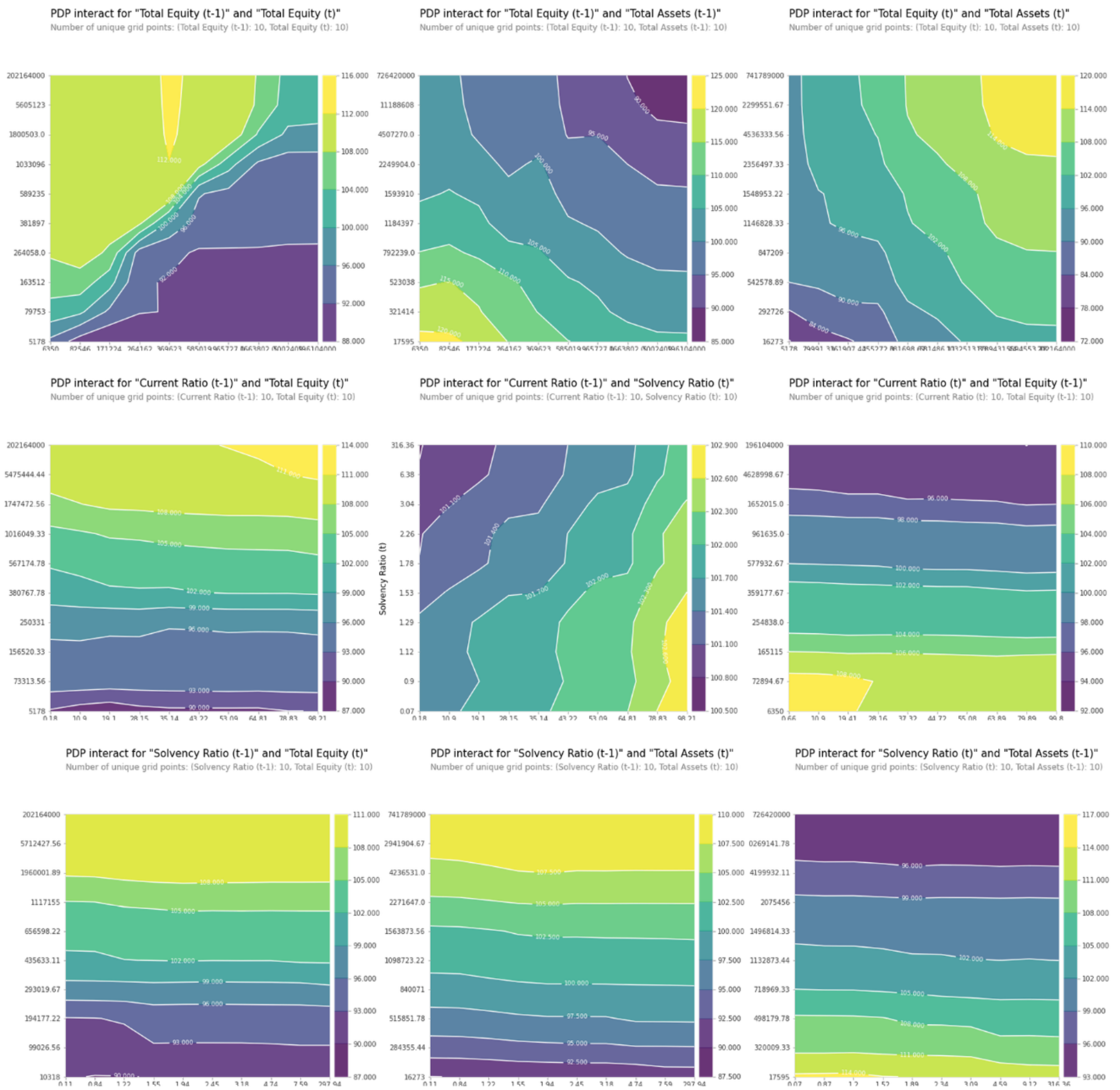

The series of visualizations above shows the two-way partial dependence plots (PDPs) for 15 most important independent variables in the Random Forest and their three MFI features. The graphs show the predicted values of the Random Forest for ten values of both independent variables. Higher values of firm growth are marked in yellow, and lower values in purple. For example, in the Total Assets (*t*) versus Working Capital (*t*) graph, we can see that firm growth is higher when Total Assets (*t*) are small and Working Capital (*t*) is high. The two-way PDP can help to identify interaction effects, but is prone to the same assumptions as the one-way PDP. As such, interpretation for our dataset is not straightforward, given the presence of significant correlations, as illustrated in the correlation matrix.

8. Evidence hierarchy

The Feature Importances, Contributions, Most Frequent Interactions, one-way partial dependences plots, and two-way partial dependence plots are different ways to explore the model learned by the Random Forest Analysis. The interpretation of these explanation methods should be done with care as each method has its own purpose, but is also subject to one or more weaknesses. Since our current aim is to use the Random Forest as part of a co-ductive reasoning process, we put more weight to those explanation methods that cover the entire dataset and use the full Random Forest. More specifically, the Feature Importances and Contributions are computed across all trees in the Random Forest and the entire dataset. We thus put most weight on these two explanations methods. The Most Frequent Interaction explanation method is computed for a subset of data for which the Random Forest has an absolute percentage error smaller than 10%. Since this is a subset of data, we rank it just below the Feature Importance and Contribution methods, but above the partial dependence plots. The one-way partial dependence plots and two-way partial dependence plots are computed for a random subset of 900 and 20 data point, respectively. As such, we treat these explanation methods as merely indicative and rank them lowest in our evidence hierarchy.

9. Structural equation models

H2. The effect of Egocentrism on firm growth runs through the positive association of Egocentrism with Operational Excellence.

lval	op	rval	Estimate	Est. Std	Std. Err	z-value	p-value
OperationalExcellence	~	Egocentrism	0.088	0.187	0.006	15.384	0.000
OperationalExcellence	~	TotalAssetstmin1	0.000	0.001	0.000	0.024	0.981
OperationalExcellence	~	WorkingCapitaltmin1	0.000	-0.001	0.000	-0.066	0.948
OperationalExcellence	~	CurrentRatiotmin1	0.000	0.002	0.000	0.152	0.879
OperationalExcellence	~	SolvencyRatiotmin1	0.000	-0.001	0.000	-0.067	0.946
OperationalExcellence	~	NumberofEmployeestmin1	0.000	-0.003	0.000	-0.213	0.832
Growth	~	Egocentrism	10.879	0.025	5.515	1.973	0.049
Growth	~	OperationalExcellence	2.141	0.002	11.749	0.182	0.855
Growth	~	TotalAssetstmin1	0.000	0.022	0.000	1.633	0.102
Growth	~	WorkingCapitaltmin1	0.000	-0.020	0.000	-1.463	0.143
Growth	~	CurrentRatiotmin1	0.001	0.028	0.000	2.297	0.022
Growth	~	SolvencyRatiotmin1	0.002	0.025	0.001	1.999	0.046
Growth	~	NumberofEmployeestmin1	-0.010	-0.028	0.004	-2.301	0.021
OperationalExcellence	~~	OperationalExcellence	0.000	0.965	0.000	57.241	0.000

H3. The effect of Egocentrism on firm growth is positively moderated by Operational Excellence.

Lval	op	rval	Estimate	Est. Std	Std. Err	z-value	p-value
Growth	~	Egocentrism	7.552	0.017	5.980	1.263	0.207
Growth	~	OperationalExcellence	-19.230	-0.021	18.776	-1.024	0.306
Growth	~	EgocentrismOperationalExcellence	198.218	0.031	135.771	1.460	0.144
Growth	~	TotalAssetstmin1	0.000	0.006	0.000	0.329	0.742
Growth	~	WorkingCapitaltmin1	0.000	-0.039	0.000	-1.741	0.082
Growth	~	CurrentRatiotmin1	0.001	0.028	0.000	2.296	0.022
Growth	~	SolvencyRatiotmin1	0.002	0.025	0.001	1.995	0.046
Growth	~	NumberofEmployeestmin1	-0.011	-0.029	0.004	-2.367	0.018

H4. The effect of Egocentrism on firm growth runs through the positive association of Egocentrism with Operational Excellence and is moderated by Operational Excellence.

lval	op	rval	Estimate	Est. Std	Std. Err	z-value	p-value
**OperationalExcellence**	~	**Egocentrism**	0.090	0.191	0.006	15.738	0.000
**OperationalExcellence**	~	**TotalAssetstmin1**	0.000	0.001	0.000	0.023	0.982
**OperationalExcellence**	~	**WorkingCapitaltmin1**	0.000	-0.001	0.000	-0.065	0.948
**OperationalExcellence**	~	**CurrentRatiotmin1**	0.000	0.002	0.000	0.151	0.880
**OperationalExcellence**	~	**SolvencyRatiotmin1**	0.000	-0.001	0.000	-0.065	0.948
**OperationalExcellence**	~	**NumberofEmployeestmin1**	0.000	-0.003	0.000	-0.222	0.824
**Growth**	~	**Egocentrism**	8.503	0.019	5.960	1.427	0.154
**Growth**	~	**OperationalExcellence**	2.044	0.002	11.757	0.174	0.862
**Growth**	~	**EgocentrismOperationalExcellence**	89.883	0.014	84.947	1.058	0.290
**Growth**	~	**TotalAssetstmin1**	0.000	0.022	0.000	1.636	0.102
**Growth**	~	**WorkingCapitaltmin1**	0.000	-0.020	0.000	-1.464	0.143
**Growth**	~	**CurrentRatiotmin1**	0.001	0.028	0.000	2.296	0.022
**Growth**	~	**SolvencyRatiotmin1**	0.002	0.025	0.001	1.998	0.046
**Growth**	~	**NumberofEmployeestmin1**	-0.010	-0.028	0.004	-2.289	0.021

10. Marginal effects plots

Marginal effects plot for H3

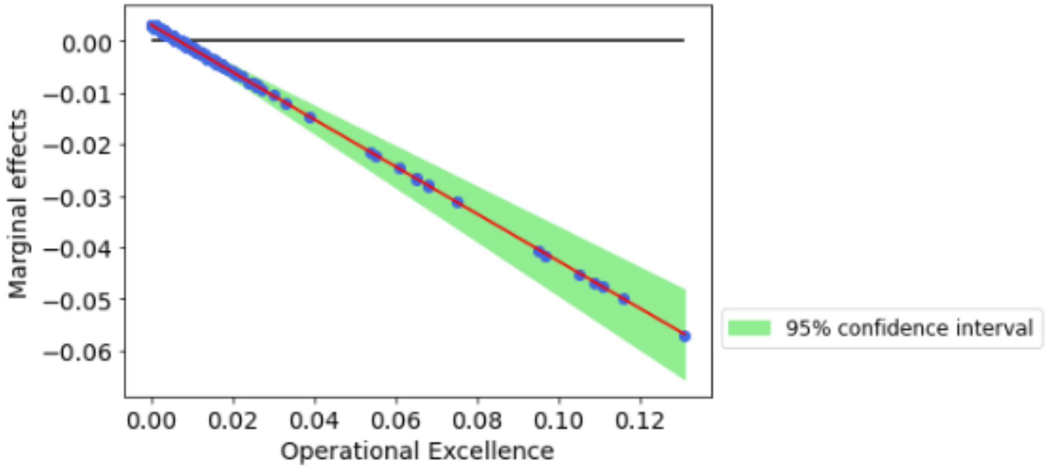

Marginal effects plot for H4

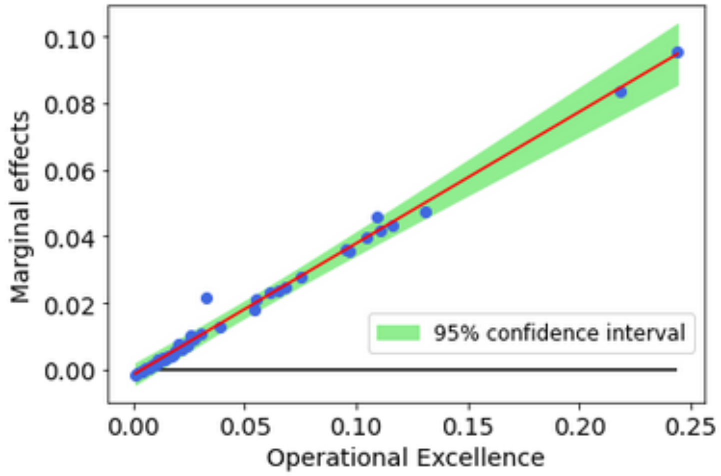

Miscellaneous

Software

The analyses for this paper were conducted in Python 3.7.0 with the following libraries: Gensim (3.2.0), PDPbox (2.0), PyCEbox (0.0.1), scikit-learn (0.23.1), treeinterpreter 0.2.2, eli5 (0.10.1), and semopy (2.3.9).

Approach

We scraped "About Us"-pages of company websites in 2016. After discarding empty pages, we ended up with 8,163 firms with valid pages. We then collected panel data on financial indicators for those firms for the years 2016-2017. We used this dataset for the machine learning part (Steps 1 to 3 of the co-duction cycle) of the paper. We divided the data in a training and test or validation set with an 80% - 20% split. The training data was used for hyper-parameter tuning and model training. The models were evaluated against the test set to get a sense of how well they would generalize to new data. After having selected the best hyper-parameters, we retrained the Random Forest on the complete dataset and then computed the explanatory outputs included in the paper. For the deductive part of the paper, we re-scraped the "About Us"-pages in 2017 and added data on financial indicators for 2018. The regression analyses and structural equation models were done on this new dataset that was not used in the machine learning part of the analysis, and which is thus unseen by the Random Forest.

Future research

New machine learning techniques and procedures have been developed since we started our initial analyses. Below, we briefly refer to a few notable examples that can be taken on board in future research.

Innovations for topic model evaluation metrics: We used perplexity to evaluate our topic model, but this has been shown to correlate weakly with human evaluations of topics. Instead, topic models now often use coherence metrics as an alternative (see [10]).

Novel topic modeling techniques: While we used unigrams as the basis for our topic modelling, new techniques permit the use of contextual word embeddings (see [11]) in combination with LDA [12].

New explanation methods: The assumption of independence between features is a major weakness of the PDP. Accumulated Local Effects (ALE) plots are an alternative that is not prone to this assumption [13].

Such innovations can be used within our co-duction cycle by functioning as a drop-in replacement for the techniques they improve upon. Future research could focus on exploring the application of these novel techniques with the co-ductive reasoning procedure, and providing guidelines and best practices for use and interpretation of these novel techniques within the inductive analysis part of the cycle.

References

1. Wallach HM, Murray I, Salakhutdinov R, Mimno D. Evaluation methods for topic models. InProceedings of the 26th annual international conference on machine learning 2009 Jun 14 (pp. 1105-1112).
2. Bergstra J, Bengio Y. Random search for hyper-parameter optimization. Journal of machine learning research. 2012 Feb 1;13(2).
3. Krstajic D, Buturovic LJ, Leahy DE, Thomas S. Cross-validation pitfalls when selecting and assessing regression and classification models. Journal of cheminformatics. 2014 Dec;6(1):1-5.
4. Breiman L. Random forests. Machine learning. 2001 Oct;45:5-32.
5. Friedman JH. Greedy function approximation: a gradient boosting machine. Annals of statistics. 2001 Oct 1:1189-232.
6. Molnar C. *Interpretable Machine Learning*. 2020. [accessed 1 October 2021] Available online: https://christophm. github.io/interpretable-ml-book.
7. Goldstein A, Kapelner A, Bleich J, Pitkin E. Peeking inside the black box: Visualizing statistical learning with plots of individual conditional expectation. journal of Computational and Graphical Statistics. 2015 Jan 2;24(1):44-65.
8. Palczewska A, Palczewski J, Robinson RM, Neagu D. Interpreting random forest models using a feature contribution method. In2013 IEEE 14th International Conference on Information Reuse & Integration (IRI) 2013 Aug 14 (pp. 112-119). IEEE.
9. Petkovic D, Altman R, Wong M, Vigil A. Improving the explainability of Random Forest classifier - user centered approach. Pac Symp Biocomput. 2018;23:204-215.
10. Mimno D, Wallach H, Talley E, Leenders M, McCallum A. Optimizing semantic coherence in topic models. InProceedings of the 2011 conference on empirical methods in natural language processing 2011 Jul (pp. 262-272).
11. Devlin J, Chang MW, Lee K, Toutanova K. Bert: Pre-training of deep bidirectional transformers for language understanding. arXiv preprint arXiv:1810.04805. 2018 Oct 11.
12. Peinelt N, Nguyen D, Liakata M. tBERT: Topic models and BERT joining forces for semantic similarity detection. InProceedings of the 58th annual meeting of the association for computational linguistics 2020 Jul (pp. 7047-7055).
13. Apley DW, Zhu J. Visualizing the effects of predictor variables in black box supervised learning models. Journal of the Royal Statistical Society Series B: Statistical Methodology. 2020 Sep;82(4):1059-86.
