# Supplementary material for: Data science and automation in the process of theorizing: Machine learning’s power of induction in the co-duction cycle
Source: PLoS One. 2024 Nov 4;19(11):e0309318. doi: 10.1371/journal.pone.0309318 (PMC11534228; doi:10.1371/journal.pone.0309318)
Supplement: S1 Data — (PDF) [file pone.0309318.s002.pdf]

Individual Application to use the

Anonymized Company Information Netherlands and Belgium dataset

I ________________________, a person engaging in research and development and a member of, consultant to, or person providing service to the following organization:

Organization:____

Corporation/Partnership/Legal Entity: ____

Postaddress:____

Telephone:____

E-mail: ____

apply to use the information designated as the Anonymized Company Information Netherlands and Belgium dataset subject to the following understandings, terms and conditions. These understandings, terms and conditions apply equally to all or to part of the information.

1. **Permitted Uses**
    a. The information may only be used for research.
    b. Small excerpts of the information may be displayed to others or published in a scientific or technical context, solely for the purpose of describing the research and development and related issues. Any such use shall not infringe on the rights of any third party including, but limited to, the authors and publishers of the excerpts.

2. **Access to the Information by Individuals**
    a. Access to the information by an individual is to be controlled by that person's organization. The organization may only grant access people working under its control; that is, its own members, consultants, or individuals providing service to the organization.
    b. Individuals may be allowed access to the information only after completion and submission of this form. The access is to be terminated when the conditions of the application no longer apply. The organization will retain the applications of all persons ever granted access to the information and make them available upon request to any of the copyright holders and to the institution or agency holding this completed application.
    c. The organization will maintain a list of people with current and recently-terminated access to the information.
    d. An individual with access may only display the information to or share the information with persons whom their organization lists as having access to the information.

3. **Ethical Use**
    a. All users agree to abide by ethical standards in their use of the information and to use best practices in data handling and storage.

4. **Liability**
    a. Any misuse of the data, including but not limited to unauthorized sharing, use for non-research purposes, or violation of the ethical use clause, may result in legal liability and revocation of access privileges.

5. **Procedure for revoking access**
    a. Access may be revoked at any time by the organization, at its discretion. Any individual whose access is revoked is required to immediately cease all use of the information and delete all copies

6. **Copyright and database rights**
    a. The copyright and database rights holders retain ownership and reserve all rights pertaining to the use and distribution of the information.
    b. Except as specifically permitted above and as necessary to use and maintain the integrity of the information on computers used by the organization; the display, reproduction, transmission, distribution or publication of the information is prohibited. Violations of the copyright restrictions on the information may result in legal liability.

By the Individual:

Signature:____

Date:____

Name:____

Title: ____

Accepted by the organization:

Signature____

Date ____

Name:____

Title: ____

Institution/

Agency:____

Please print, complete, scan, and e-mail this form to d.a.kolkman@tue.nl, research@jads.nl or supportoffice.ads.ics@uu.nl.
